# Dynamic emotional expressions do not modulate responses to gestures

**DOI:** 10.1016/j.actpsy.2020.103226

**Published:** 2021-01

**Authors:** Harry Farmer, Raqeeb Mahmood, Samantha E.A. Gregory, Polina Tishina, Antonia F. de C. Hamilton

**Affiliations:** a School of Human Sciences, University of Greenwich, United Kingdom; b Institute of Lifecourse Development, University of Greenwich, United Kingdom; c Institute of Cognitive Neuroscience, University College London, United Kingdom; d Department of Psychology, University of Bath, United Kingdom; e School of Life & Health Sciences, Aston University, United Kingdom

**Keywords:** Meaningful gestures, Automatic imitation, Emotion, Stimulus response compatibility, Facial expressions

## Abstract

The tendency to imitate the actions of others appears to be a fundamental aspect of human social interaction. Emotional expressions are a particularly salient form of social stimuli (Vuilleumier & Schwartz, 2001) but their relationship to imitative behaviour is currently unclear. In this paper we report the results of five studies which investigated the effect of a target's dynamic emotional stimuli on participants' tendency to respond compatibly to the target's actions. Experiment one examined the effect of dynamic emotional expressions on the automatic imitation of opening and closing hand movements. Experiment two used the same basic paradigm but added gaze direction as an additional factor. Experiment three investigated the effect of dynamic emotional expressions on compatibility responses to handshakes. Experiment four investigated whether dynamic emotional expressions modulated response to valenced social gestures. Finally, experiment five compared the effects of dynamic and static emotional expressions on participants' automatic imitation of finger lifting. Across all five studies we reliably elicited a compatibility effect however, none of the studies found a significant modulating effect of emotional expression. This null effect was also supported by a random effects meta-analysis and a series of Bayesian *t*-tests. Nevertheless, these results must be caveated by the fact that our studies had limited power to detect effect sizes below d = 0.4. We conclude by situating our findings within the literature, suggesting that the effect of emotional expressions on automatic imitation is, at best, minimal.

## Highlights

• The effect of emotional expressions on automatic imitation is currently unclear.
• We used dynamic expressions to investigate this effect across five experiments.
• We found that the expressions did not affect the imitation of response to gestures.
• This was the case even when the gestures had affective or social meaning.

## 1 Introduction

The tendency to imitate the actions of others appears to be a fundamental aspect of human social interaction. In humans imitation can be seen across a range of modalities and behaviours including the imitation of hand gestures (Press, Bird, Walsh, & Heyes, 2008; Stürmer, Aschersleben, & Prinz, 2000), motion kinematics (Forbes, Suddell, Farmer, Logeswaran, & Hamilton, 2019; Krishnan-Barman, Forbes, & de C Hamilton, 2017), facial expressions (Hess & Fischer, 2013; Seibt, Mühlberger, Likowski, & Weyers, 2015) and vocalisations (Pickering & Garrod, 2013). Cross-species research suggests that humans imitate across a wide range of tasks and domains with higher fidelity and greater sensitivity to context than any other species (Subiaul, 2016; Whiten, 2011).

This evidence for the range and specificity of human imitation raises important questions regarding its origins (Cook, Bird, Catmur, Press, & Heyes, 2014; Heyes, 2009) and function (Farmer, Ciaunica, & de C Hamilton, 2018). One of the most established theories on the function of imitation proposes that imitation acts as a social glue which is strategically deployed in order to build affiliation with others (Chartrand & Bargh, 1999; Lakin, Jefferis, Cheng, & Chartrand, 2003; Wang, & Hamilton, A. F. de C., 2012). In support of this theory is evidence that being imitated can lead to positive appraisals of the imitator (Dignath, Lotze-Hermes, Farmer, & Pfister, 2018; Lakin et al., 2003) and conversely, that people imitate a target more when that target is viewed more positively (Blocker & McIntosh, 2016; Likowski, Mühlberger, Seibt, Pauli, & Weyers, 2008; Stel et al., 2010).

Further evidence for the affiliative function of imitation comes from findings that the tendency to imitate can be modulated by a wide range of social cues including: motivation to affiliate (Lakin, Chartrand, & Arkin, 2008; Over & Carpenter, 2009; Watson-Jones, Whitehouse, & Legare, 2015); group membership (Bourgeois & Hess, 2008; Losin, Iacoboni, Martin, Cross, & Dapretto, 2012; Yabar, Johnston, Miles, & Peace, 2006); and the imitation target's attractiveness (Karremans & Verwijmeren, 2008; van Leeuwen, van Baaren, Martin, Dijksterhuis, & Bekkering, 2009).

Before reviewing the literature further, it is worth noting that there are several forms of imitative behaviour. Farmer, Carr, Svartdal, Winkielman, and de C Hamilton (2016) identify three common forms of imitation in the literature. The first of these is behavioural mimicry, the tendency of people to naturally copy others' movements during social interactions, which is usually studied via naturalistic observation paradigms. The second is facial mimicry, the tendency of people to (overtly or covertly) imitate the facial movements and expressions of others. The third is automatic imitation (AI) which is the form of imitation investigated in the current paper. AI can be thought of as a particular form of stimulus-response compatibility effect (SRC) in which an action stimulus is paired with the same action response (congruent) or a different action response (incongruency). By comparing participants' reaction times (RTs) when responding with similar vs different responses across a range of trials it is possible to derive a congruency effect which can act as a quantitative measure of the strength of imitative tendency (Brass, Bekkering, Wohlschläger, & Prinz, 2000; Heyes, 2011).

While AI lacks the ecological validity of behavioural mimicry studies it allows for greater experimental control and standardisation of stimuli. It is currently unclear how closely AI and more naturalistic measures of mimicry are related. One recent study found no correlation between the two forms of imitation (Genschow et al., 2017) and a neuroimaging study suggested they depend on dissociable neural systems (Hogeveen et al., 2014) while a developmental study suggested a link between AI and experience of behavioural synchrony (O'Sullivan, Bijvoet-van den Berg, & Caldwell, 2018). Despite this uncertainty a recent meta-analysis (Cracco et al., 2018) found strong evidence that AI is a robust and largely automatic process that can be modulated by a range of factors including: action goals; how closely the stimuli physically resemble a human and the extent to which the gender of the actor and the outcome of the observed action overlapped with the gender of the imitator and the outcome of the executed action.

In addition to these meta-analytic findings there is evidence that more explicitly social factors can modulate AI. For example, a number of studies have shown an effect of gaze on AI with a stronger congruency effect when observing a model making direct, compared to averted eye contact (Forbes, Wang, & Hamilton, 2016; Wang & de C Hamilton, 2014; Wang, Newport, & Hamilton, 2011). Other studies have demonstrated that the congruency effect is increased when participants have been primed with pro-social sentences relating to the self (Leighton, Bird, Orsini, & Heyes, 2010; Wang & de C Hamilton, 2013). Interestingly neuroimaging studies have suggested that both of these effects are driven by activity in the medial pre-frontal cortex (Wang & Hamilton, 2015; Wang, Ramsey, & de C Hamilton, 2011), an area of the brain heavily implicated in processing other's mental states and other aspects of social cognition (Van Overwalle, 2009). Other studies have found that the strength of interpersonal relationship between actor and imitator (Maister & Tsakiris, 2016) and group identity (Gleibs, Wilson, Reddy, & Catmur, 2016; Rauchbauer, Majdandžić, Hummer, Windischberger, & Lamm, 2015; Rauchbauer, Majdandžić, Stieger, & Lamm, 2016) can also modulate AI. However, it should be noted that other socially relevant factors such as social status and power do not appear to modulate AI (Farmer et al., 2016).

Emotional expressions are a particularly salient form of social stimuli (Vuilleumier & Schwartz, 2001) which are processed rapidly and without conscious awareness (Batty & Taylor, 2003; Smith, 2012) and have been shown to interfere with non-imitative motor responses (Renard, de Jong, & Pijnenborg, 2017; Seidel, Habel, Kirschner, Gur, & Derntl, 2010). Emotional expressions are closely linked to the phenomena of facial mimicry discussed above, with considerable evidence that participants will automatically imitate the emotional expressions of others (Dimberg, Thunberg, & Elmehed, 2000; Hess & Fischer, 2014). However, in the current study we are interested in emotional expressions primarily due to their power as a form of social signalling (Frith, 2009; Hareli & Hess, 2012). If theories that claim a function of imitation is the creation or maintenance of social bonds (e.g. Chartrand & Bargh, 1999; Lakin et al., 2003; Wang, & Hamilton, A. F. de C., 2012) are correct then it might be expected that the social signals given by emotional expressions would have a modulating effect on imitative tendencies as measured via automatic imitation. More specifically it might be expected that people would be more likely to imitate when observing positive expressions indicating affiliative intent than when observing neutral or negative expressions.

A number of previous studies have investigated the effect of different emotional expressions on the AI of finger movements (Butler, Ward, & Ramsey, 2016; Crescentini, Mengotti, Grecucci, & Rumiati, 2011; Grecucci et al., 2013; Rauchbauer et al., 2015, Rauchbauer et al., 2016). However, to date the results of these studies have been somewhat inconclusive. Crescentini et al. (2011) found no evidence that either angry or sad expressions led to a change in congruency effect compared to neutral expression and Grecucci et al. (2013) have a similar null result when comparing fearful faces to neutral expressions. By contrast Racuhbauer and colleagues found greater AI for happy compared to angry faces (Rauchbauer et al., 2015) but that this effect was modulated by racial group with greater AI when viewing an angry outgroup face (Rauchbauer et al., 2016). Finally Butler et al. (2016) compared happy, angry and neutral expressions but found only limited evidence for an effect of emotional expressions on congruency effects and even then only for the happy vs neutral expression. They also conducted a meta-analysis of all previous data which suggested no strong differences between angry and happy expressions or angry and neutral expressions on congruency effects and only a weak effect of happy vs neutral expressions. Given these unclear results and the ongoing concerns regarding the reproducibility of results in cognitive science (Shrout & Rodgers, 2018) we sought to further explore the effect of emotions on AI.

One limitation of previous studies of the impact of emotional expressions on AI is that they relied on the use of static images of often exaggerated expressions. Ferreira-Santos (2015) argues that emotion expression stimuli can be considered on a continuum between experimental control and ecological validity with schematic diagrams at one end of the spectrum, live actors at the other and static photographs and videos in the middle. Research on the use of dynamic compared to static emotional expression stimuli has found that dynamic stimuli leads to enhanced emotional arousal (Sato & Yoshikawa, 2007), emotion recognition (Wehrle, Kaiser, Schmidt, & Scherer, 2000) and many other processing advantages (see Krumhuber, Kappas, & Manstead, 2013 for a detailed review). However, to date it is unclear whether such dynamic facial expressions can modulate AI.

The current study sought to fill this gap by investigating whether different dynamic emotional expressions modulated SRC via a series of experimental studies. Experiment one investigated the extent to which genuine and polite smiles, frowns and neutral expressions modulated the imitation of intransitive motor actions. Experiment two used a similar stimulus set but also investigated the extent to which the gaze of the imitation target modulated response. Experiment three examined the same set of emotional expressions but rather than using a traditional AI paradigm examined the effect of emotional expression on the more explicitly social SRC of a handshake. Experiment four returned to examining the effect of emotional expression on AI but this time in the context of meaningful and valenced social gesture (the thumbs up and middle finger signs) and with only the more clearly valenced genuine smile and frown expressions. Finally experiment five compared the effect of our dynamic emotional expression stimuli with the static images used in previous studies investigating emotional expressions and AI in the context of finger lifting.

## 2 Experiment 1: does emotional expression modulate automatic imitation?

### 2.1 Experiment 1: introduction

Experiment one investigated the effect of emotional expressions on the AI of the intransitive actions of hand opening and closing. As detailed above, previous studies have found that this form of AI can be modulated by the socially relevant factors of pro-social priming and gaze. In addition these actions are not goal directed and do not have a clear difference in spatial location meaning that they are not susceptible to being confounded by either effector or spatial matching effects (Heyes, 2011) and so act as a good measure of “pure” AI.

One plausible mechanism for the previously reported increase in imitation towards happy faces (Rauchbauer et al., 2015) is that participants view the happy face as a signal of affiliative intent and that this pro-social cue acts to increase their tendency to give an imitative response (Chartrand & Lakin, 2013). However, not all smiling faces convey the same affiliative social signal. Duchenne (1990) distinguished between genuine and polite smiles with genuine smiles involving not only the pulling up of the lip corners by the *zygomaticus major* muscle but also the lifting of the cheeks and narrowing of the eyes by the *orbicularis oculi* muscle. The use of this latter muscle is more difficult, although not impossible (Krumhuber & Manstead, 2009), to consciously control meaning that, at least among Western populations (see Thibault, Levesque, Gosselin, & Hess, 2012), it is seen as a sign of genuine, as opposed to feigned, enjoyment. Research has demonstrated that participants can reliably distinguish between genuine and polite smiles, with genuine smiles being viewed as more reflective of genuine happiness (Gosselin, Perron, Legault, & Campanella, 2002), being a more valuable form of social reinforcement (Shore & Heerey, 2011), indicating less psychological distance (Bogodistov & Dost, 2017) and leading to more positive person judgements (Johnston, Miles, & Macrae, 2010; Quadflieg, Vermeulen, & Rossion, 2013) than polite smiles. Of particular interest for the current study is the finding that genuine smiles lead to stronger and more rapid facial mimicry than polite smiles, even in the *zygomaticus major* which is involved in the production of both expressions (Heerey & Crossley, 2013; Krumhuber, Likowski, & Weyers, 2014).

If it is the case that the increased AI found for smiles as compared to neutral expressions or frowns is due to it signalling increased affiliative intent then it is plausible that this effect will only be found when participants view what they perceive as a genuine as opposed to a polite smile. To test this hypothesis we included both genuine and polite smiles in our emotional expression stimuli for experiment one along with a frown and a neutral expression, which matched the other expressions examined by Butler et al. (2016). Based on the idea that AI is modulated by signals of affiliative intent and based on previous ratings of the valance of our emotional expressions (see below) we predicted that participants would show the greatest congruency effects when the observed gestures were paired with genuine smiles, followed by polite smiles, neutral expressions and finally frowns.

### 2.2 Experiment 1: methods

#### 2.2.1 Participants

Here and in all other experiments, participants were recruited from the Institute of Cognitive Neuroscience database and were paid for taking part in the experiment. All participants had normal or corrected to normal vision and gave informed consent to participate. 32 participants (16 males) took part in this experiment. One male participant was excluded from the final analysis as they had less than 85% valid RTs in their non-baseline trials leaving a final sample of 31 participants with a mean age of 25.5 (SD = 5.46). A sensitivity analysis carried out using G*Power (Faul, Erdfelder, Lang, & Buchner, 2007) indicated that this sample size was sufficient to detect an effect of emotional expression on percentage congruency effect scores (PCongE) with an effect size (*f)* of 0.21 with alpha set at 0.05 and beta set at 0.8 (see Supplementary Materials 1.1 for full protocol).

#### 2.2.2 Design and procedure

This study assessed the impact of four different dynamic emotional expressions on participants' tendency to imitate the observed actions on the screen, using an AI task. The experiment was designed as a 2 (Actor Gender: female, male) x 4 (Emotional Expression: genuine smile, polite smile, neutral, frown) x 2 (Congruency of Observed and Executed Actions: congruent, incongruent) within-subjects design. The actions used were a hand opening vs a hand closing. The dependent variables were reaction times (RTs) and the percentage congruency effect. Participants were first given an information sheet and asked to give written consent for their participation. They were then given a verbal and written explaining the real purpose of the experiment.

#### 2.2.3 Materials

##### 2.2.3.1 Stimuli preparation

In order to generate the stimuli, four male and four female actors were filmed making the four different emotional expressions: genuine smiles; polite smiles; frowns; and neutral expressions. In each video the actor started with their head looking down before looking up into the camera and making the appropriate expression. The actors were instructed to make naturalistic rather than exaggerated expressions and were given short vignettes telling them to imagine being at a party and seeing a friend (Genuine Smile), someone they did not like but had to be polite to (Polite Smile) or someone who they were angry with (Frown). Each actor filmed several clips for each emotional expression which were cut to a length of 2520 ms. Then, two clips of each expression from the same actor were selected by the researchers and these were used for a pilot study.

**Fig. 1:**
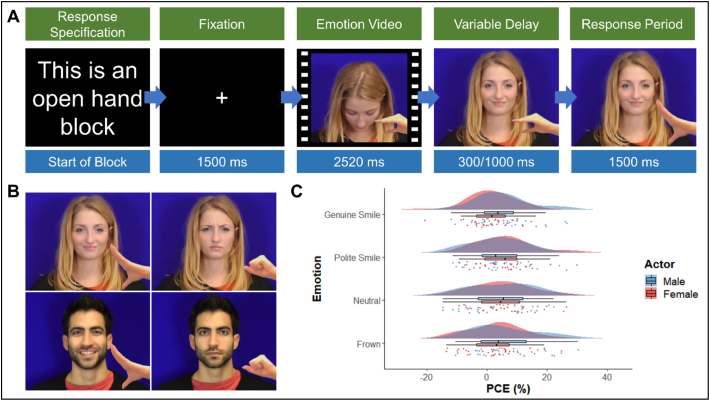
A) Time course of a congruent trial showing specification of response at start of block and then the four time periods within each trial. B) Examples of female polite smile open hand, female frown closed hand, male genuine smile open hand and male neutral closed hand stimuli. C) Raincloud plot showing mean PCongE across emotion and actor. Clouds represent distribution, raindrops represent individual datapoints.

Independent ratings were given to these clips by 20 participants to assess the validity of the emotional expressions for valence and arousal. The final stimuli were chosen based on their Likert scale intensity ratings (i.e., how much the emotion subjectively aroused participants), genuine ratings (how real the emotion displayed was) and positivity ratings (i.e., how positive and pleasant the emotion was). Thus, a final selection was made of one video clip for each emotion from the overall best male and overall best female actor. These clips were the most appropriately rated emotionally valid clips for each emotion on the three dimensions and were also closely matched in ratings between actors (see Supplementary Materials for the mean ratings of the clips used in each of the individual experiments). Two different actors, one from each gender, were purposefully selected instead of using one actor's expressions as all stimuli, because a previous study reported increases in mimicry towards attractive opposite sex targets (van Leeuwen, Veling, van Baaren, & Dijksterhuis, 2009). Hand stimuli from Wang, Newport, and Hamilton (2011) were then overlaid onto these videos using a custom Matlab script. A baseline stimuli of a hand midpoint between opening and closing was added to each frame of the video, then the final frame of the video was taken and three additional images of the hand moving to its final position were added to create stimuli for the hand open and hand closed conditions (See Fig. 1B for example stimuli).

##### 2.2.3.2 Apparatus

The experiment was run in MATLAB (Mathworks, 2015) using Cogent (Cogent 2000 Team & Romaya, 2015) to display the images and videos. Participants' motion data (hand opening and closing movements) were collected using two Polhemus Liberty magnetic motion trackers which were attached to the thumb and middle finger of the participant's right hand with medical tape. The motion tracking data were fed from the Polhemus Liberty Device (Polhemus, 2012) into MATLAB using the programme Autrak (AuSim, 2010) with an effective sampling rate of 240 Htz. The task was presented to the participants on a projector screen so that they were approximately life sized to simulate a more valid social interaction.

##### 2.2.3.3 Automatic imitation task

The task consisted of 12 blocks each of which contained 16 experimental trials plus an initial trial which was discarded during analysis. In addition, 32 baseline trials in which the actor's hand did not move were split among the blocks (Eight blocks had three baselines and four blocks had two). Thus, a participant ran through a total of 236 trials. At the start of each block participants were given an on-screen instruction to make the same pre-specified executed movement (either open or closed hand) as soon as the actor's hand began to move. They were instructed to do this as quickly and as accurately as possible, regardless of what the actor's movements were. In the case of baseline trials participants were instructed not to make the pre-specified movement. The pre-specified action switched between each block and the starting order was counterbalanced between participants. The identity of the actor switched every 2 blocks and the order of actor was counterbalanced across participants. The emotion and observed action were randomised across trials in each block. Prior to starting the main task participants completed an additional practice block with a different female actor which consisted of ten trials.

Participants began each trial with their elbow rested upright on the desk and in a semi-open hand position. In each trial a fixation cross was presented at centre for 1500 ms then the video clip of the emotional expression was played with a length of 2520 ms. Following this the actor's hand moved into either an open or closed position (experimental trials) or remained in the same position (baseline trials). To prevent anticipation effects there was a delay of either 300 ms or 1000 ms before the actor's hand moved, these timings were similar to those used in previous studies that probed the impact of social stimuli on automatic imitation (Leighton et al., 2010; Press, Gillmeister, & Heyes, 2007). The hand movement consisted of 3 frames with a delay between them of 80 ms. Response times were recorded from the beginning of the actor's hand movement, and participants had 1500 ms in which to make a response. The total trial length was either 5820 ms or 6520 ms dependent upon the time delay of the start of the actor's hand movement (see Fig. 1A).

#### 2.2.4 Data processing and analysis

Of the 236 trials for each participant, the first trial from each block along with the 32 baseline trials were removed leaving a total of 192 experimental trials, 12 per condition. For each experimental trial, the timing and position data in the X, Y and Z axes for both trackers were extracted from the MATLAB output. Hand aperture was calculated as the distance between thumb and finger markers. Mean aperture velocity across the three axes was calculated and then smoothed with a 17 ms window. Peak velocity was defined as the first peak in the velocity profile which reached at least one third of the largest peak. This allowed the exclusion of rare “wobbles” in the data and to pick the initial fast hand opening or closing movement. Graphs depicting the tangential velocity and the velocity of each marker were then generated and manually checked to avoid any motion artefacts and to remove error trials in which the participant made the incorrect action. Reaction times (RTs) were calculated as the time from the presentation of the first frame of the hand movement to the time when the participant's hand aperture reached its first peak open or close velocity.

Once all trials had been processed, mean RTs for each condition (Actor x Emotion x Congruence) were calculated. Error trials (0.80% of experimental trials) and trials with RTs less than 50 ms or greater than 1000 ms were excluded from this analysis (1.92% of experimental trials) were excluded from the analysis. Participants who had less than 85% valid experimental trials were not included in the final analysis (one participant).

Previous research has demonstrated that variance in overall mean RT is a major predictor of the compatibility effect (Butler, Ward, & Ramsey, 2015), therefore RTs for each condition were converted into percentage congruence effects (PCongEs) using the following equation taken from Forbes et al. (2016):

$$\mathrm{PCongE}=\frac{\mathrm{MeanIncongruentRT}-\mathrm{MeanCongruentRT}}{\mathrm{MeanOverallRT}}\times 100$$

### 2.3 Experiment 1: results

Two repeated measures ANOVAs were conducted on the AI data (see Supplementary Materials 1.3 for an analysis of accuracy scores). The first took raw RTs as the DV and actor, emotion and congruency as the IVs. There was no significant effect of actor, *F*(1,30) = 0.12, *p =* .737, pƞ^2^ = 0.004. A significant effect of emotion was found, *F*(3, 90) = 14.81, *p* < .001, pƞ^2^ = 0.330. Bonferroni corrected pairwise comparisons of the estimate marginal means (EMMs) indicated that this effect was due to a significantly slower response time in the neutral condition (EMM = 397.29, standard error (SE) = 16.65) than in the genuine smile (EMM = 379.54, SE = 16.15, *p* < .001, *d* = −0.194), polite smile (EMM = 384.51, SE = 16.22, *p <* .001, *d* = −0.140) and frown (EMM = 382.29, SE = 16.65, *p* < .001, *d* = −0.162) conditions. There were no significant differences found between the three other emotional expressions. A significant effect of congruency was also found, *F*(1, 30) = 22.99, *p* < .001, pƞ^2^ = 0.434, due to participants being faster to respond in the congruent condition (EMM = 376.14, SE = 14.86) compared to the incongruent condition (EEM = 395.63, SE = 17.91). There was no significant interaction between actor and emotion *F*(3, 90) = 1.31, *p* = .277, pƞ^2^ = 0.042; between actor and congruency, *F*(1, 30) = 0.63, *p* = .436, pƞ^2^ = 0.020; or between emotion and congruency, *F*(3, 90) = 0.74, *p* = .532, pƞ^2^ = 0.024. The three-way interaction was also non-significant, *F*(3, 90) = 2.17, *p* = .098, pƞ^2^ = 0.067 (see Table 1).

**Table 1:** Means and standard deviations for congruent, incongruent and percentage congruency effects for each condition in experiment one.

Conditions		Cong (ms)		Incong (ms)		PCongE (%)	
Actor	Emotion	Mean	SD	Mean	SD	Mean	SD
Female	Genuine Smile	378.18	87.50	385.49	98.88	1.46	7.62
	Polite Smile	373.98	80.58	401.23	106.44	6.08	9.22
	Neutral	383.84	85.56	406.95	107.12	5.11	9.86
	Frown	374.59	92.55	387.04	95.84	3.11	9.19
Male	Genuine Smile	366.95	85.49	387.55	96.73	5.11	9.22
	Polite Smile	372.65	83.17	390.16	99.92	3.75	8.84
	Neutral	389.11	87.62	409.25	103.34	4.70	10.18
	Frown	369.85	81.25	397.35	113.16	5.95	10.66

The second ANOVA took PCongEs as the DV and actor and emotion as the IVs. This analysis found no significant effect of either actor, *F*(1, 30) = 0.49, *p =* .487, pƞ^2^ = 0.016; or emotion, *F*(1, 90) = 0.46, *p =* .711, pƞ^2^ = 0.015. Nor was there a significant interaction between the two IVs, *F*(3, 90) = 1.97, *p* = .124, pƞ^2^ = 0.062, (see Fig. 1C and Table 1).

### 2.4 Experiment 1: discussion

Experiment one revealed a clear congruency effect during the AI task, with faster responses on congruent trials compared to incongruent ones. In addition, we found an effect of emotion on reaction times with significantly shorter reaction times for both genuine and polite smiles and for frowns compared to for neutral facial expressions. This may be because arousing emotional stimuli have been shown to facilitate response times (Briesemeister, Kuchinke, & Jacobs, 2011; Pessoa, Kastner, & Ungerleider, 2002; Zeelenberg, Wagenmakers, & Rotteveel, 2006). Frowns are particularly highly arousing because they indicate potential threat or conflict (Carretié, Albert, López-Martín, & Tapia, 2009) whereas smiles, particularly genuine ones, can generate arousal via a pleasant feeling (Krumhuber et al., 2014). Supporting evidence for the arousal effect in this study comes from data that suggests that the genuine and polite smiles and the frown were rated as more arousing than the neutral expression by participants in our stimulus validation study (see Supplementary Materials: Table S1).

However, when testing for the effect of emotional expression on AI the key question is whether the expressions modulated the difference between congruent and incongruent trials. Both the interaction between congruency and emotion for the RTs and the main effect of emotion for the PCongE data were non-significant suggesting that emotional expressions did not affect AI.

## 3 Experiment 2: does emotional expression and gaze direction modulate automatic imitation?

### 3.1 Experiment 2: introduction

The failure to find any evidence for a significant EFFECT of dynamic emotional expression on AI in experiment one is in contrast to previous studies that examined the influence of static emotional expressions on AI (Butler et al., 2016; Rauchbauer et al., 2015, Rauchbauer et al., 2016). This finding is also surprising given research showing that other components of face processing, such as gaze direction, can affect the tendency to imitate (e.g. Forbes et al., 2016; Wang & de C Hamilton, 2014), and evidence that emotional expressions and gaze direction are closely interlinked aspects of face perception (Rigato & Farroni, 2013). An interaction between gaze and emotion appears to occur even in new born infants who have been show to prefer direct gaze for happy but not fearful or neutral expressions (Rigato, Menon, Johnson, & Farroni, 2011). In adults there is strong evidence for a bi-directional relationship with direct gaze facilitating the processing of approach oriented emotions such as anger and joy (Adams Jr. & Kleck, 2005; Ewbank, Jennings, & Calder, 2009; Sander, Grandjean, Kaiser, Wehrle, & Scherer, 2007) and happy faces being judged by participants as looking directly at them compared to angry, fearful or neutral expression (Lobmaier, Tiddeman, & Perrett, 2008). The interaction between gaze and expression has also been found to modulate affective evaluations of objects (Bayliss, Frischen, Fenske, & Tipper, 2007). Most relevantly for the current paper is evidence that gaze and emotion can interact to change motor responses and influence approach-avoidance behaviour (Ozono, Watabe, & Yoshikawa, 2012).

Given the strong links between gaze and emotion outlined above along with previous evidence that gaze direction reliably modulates AI, here in our second experiment we investigated whether the interaction between gaze direction and emotional expression led to a modulation in the AI of intransitive hand movements.

### 3.2 Experiment 2: methods

#### 3.2.1 Participants

25 participants (12 male) took part the experiment. One female participant was excluded from the final analysis as she had less than 85% valid trials leaving a final sample of 24 participants with a mean age of 24.5 (SD = 6.03). A sensitivity analysis carried out using G*Power (Faul et al., 2007) indicated that this sample size was sufficient to detect an effect of emotional expression on congruency with an effect size (*f)* of 0.24 with alpha set at 0.05 and beta set at 0.8 (see Supplementary Materials 2.1 for full protocol).

#### 3.2.2 Design and procedure

This experiment assessed the impact of dynamic emotional expressions and gaze direction on participants' tendency to imitate the observed actions on the screen, using an AI task. The experiment was designed as a 2 (Gaze Direction: direct, averted) x 4 (Emotional Expression: genuine smile, polite smile, neutral, frown) x 2 (Congruency of Observed and Executed Actions: congruent, incongruent) within-subjects design. The actions used were a hand opening vs a hand closing. The dependent variables were reaction times and the percentage congruency effect. The procedure matched experiment one .

#### 3.2.3 Materials

##### 3.2.3.1 Stimuli preparation

The emotional expression stimuli were taken from the same set of clips as used in experiment one. Due to the addition of direct and averted gaze as conditions and the fact that we found no significant difference between the male and female actors in experiment one, in this experiment the male faces were dropped and only the female faces were used. The averted gaze stimuli were produced by placing a second camera to the left of the one used to film the direct gaze and the timelines of the cameras were aligned so that the same clip was used for both direct and averted gaze. As in experiment one, hand stimuli from Wang, Newport, and Hamilton (2011) were then overlaid onto still images of the final video frame of each clip to create open, closed and neutral hand stimuli using a custom Matlab script.

##### 3.2.3.2 Apparatus

The apparatus used to present the stimuli and collect motion data for this study was the same as that used for experiment one.

##### 3.2.3.3 Automatic imitation task

**Fig. 2:**
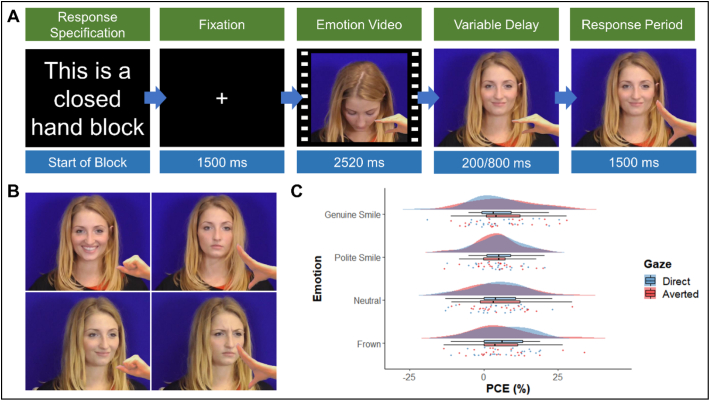
A) Time course of an incongruent trial showing specification of response at start of block and then the four time periods within each trial. B) Examples of direct genuine smile closed hand, direct neutral open hand, averted polite smile closed hand and averted frown open hand stimuli. C) Raincloud plot showing mean PCongE across emotion and gaze direction. Clouds represent distribution, raindrops represent individual datapoints.

The task consisted of 6 blocks each of which contained 32 experimental trials plus an initial trial which was discarded during analysis. In addition, 32 baseline trials in which the actor's hand did not move were split among the blocks (four blocks had five baselines and two blocks had six). Thus, a participant ran through a total of 230 trials. As with experiment one, the executed movement for each block was pre-specified prior to the start of the block and counterbalanced between blocks. The order of emotion, gaze and observed action were randomised across the block. Prior to starting the main task participants completed an additional practice block with a different female actor which consisted of ten trials. Each trial's timeline was the same as study one, however the length of the variable delay between the end of the emotional expression and the movement of the actor's hand was either 200 ms or 800 ms, these timings were based on previous studies investigating the effect of gaze on automatic imitation (Wang & de C Hamilton, 2014; Wang, Newport, & Hamilton, 2011). The total trial time was either 5720 ms or 6320 ms depending on the delay (see Fig. 2A).

#### 3.2.4 Data processing and analysis

Of the 230 trials for each participant, the first trial from each block along with the 32 baseline trials were removed leaving a total of 192 experimental trials, 12 per condition. Peak velocity, reaction time and PCongE were calculated for each condition in the same manner as in experiment one. As in experiment one, graphs depicting tangential velocity and the velocity of each marker were generated to check for motion artefacts and error trials in which participants made the wrong movement. These error trials (0.63% of experimental trials) and trials with RTs less than 50 ms or greater than 1000 ms were excluded from this analysis (4.29% of experimental trials). Participants who had less than 85% valid experimental trials were not included in the final analysis (one participant).

### 3.3 Experiment 2: results

Two repeated measures ANOVAs were conducted on the AI data (see Supplementary Materials 2.3 for an analysis of accuracy scores). The first took raw RTs as the DV and gaze, emotion and congruency as the IVs. There was no significant effect of gaze, *F*(1,23) = 0.367, *p =* .551, pƞ^2^ = 0.016. A significant effect of emotion was found, *F*(3, 69) = 6.53, *p* = .001, pƞ^2^ = 0.221. Bonferroni corrected pairwise comparisons of the estimate marginal means indicated that this effect was due to a significantly slower response time in the neutral condition (EMM = 400.39, SE = 13.58) than in the genuine smile (EMM = 383.04, SE = 13.62, *p* = .043, *d* = −0.260) and polite smile (EMM = 382.78, SE = 13.13, *p* = .005, *d* = −0.269) conditions. No other differences between the emotional expressions were significant. A significant effect of congruency was also found, *F*(1, 23) = 27.28, *p* < .001, pƞ^2^ = 0.543, due to participants being faster to respond in the congruent condition (EMM = 377.47, SE = 11.51) compared to the incongruent condition (EEM = 400.31, SE = 15.04). There was no significant interaction between gaze and emotion *F*(3, 69) = 0.55, *p* = .652, pƞ^2^ = 0.023; between gaze and congruency, *F*(1, 23) = 0.07, *p* = .796, pƞ^2^ = 0.003; or between emotion and congruency, *F*(3, 69) = 0.38, *p* = .768, pƞ^2^ = 0.016. The three-way interaction was also non-significant, *F*(3, 69) = 0.30, *p* = .824, pƞ^2^ = 0.013 (see Table 2).

**Table 2:** Means and standard deviations for congruent, incongruent and percentage congruency effects for each condition in experiment two.

Conditions		Cong (ms)		Incong (ms)		PCongE (%)	
Gaze	Emotion	Mean	SD	Mean	SD	Mean	SD
Direct	Genuine Smile	374.06	60.58	395.03	83.68	4.80	9.84
	Polite Smile	373.62	60.84	395.01	72.81	5.08	7.42
	Neutral	388.58	60.59	407.69	75.39	4.43	8.74
	Frown	377.36	62.75	404.75	82.60	6.47	8.32
Averted	Genuine Smile	367.38	52.49	395.70	82.20	6.57	10.65
	Polite Smile	371.79	55.18	390.70	77.68	4.21	7.43
	Neutral	391.80	67.70	413.47	75.35	5.21	9.37
	Frown	375.14	57.36	400.11	76.84	5.89	10.58

The second ANOVA took PCongEs as the DV and gaze and emotion as the IVs. This analysis found no significant effect of either gaze, *F*(1, 23) = 0.06, *p =* .815, pƞ^2^ = 0.002; or emotion, *F*(1, 23) = 0.34, *p =* .799, pƞ^2^ = 0.014. Nor was there a significant interaction between the two IVs, *F*(3, 69) = 0.29, *p* = .831, pƞ^2^ = 0.013, (see Fig. 2C and Table 2).

### 3.4 Experiment 2: discussion

As with experiment one, we found a strong effect of congruency but no evidence for a main effect of emotional expression or an interaction between emotional expression and gaze direction in the PCongE data. This replication of experiment one's results as regards emotions suggests that, at least for dynamic expressions, the emotional expression of a target does not interfere in the tendency to mimic that target.

Somewhat more surprisingly however we also failed to find any effect of gaze on AI despite a well-established literature finding such effects using very similar stimuli and procedures (Forbes et al., 2016; Wang, Newport, & Hamilton, 2011; Wang, Ramsey, & de C Hamilton, 2011). One explanation for our failure to find an effect in the current experiment is that, as noted above, there are strong interaction effects between gaze and emotional expression with some expressions such as smiles leading to averted gazes to be perceived as more direct (Lobmaier et al., 2008). It is therefore possible that the effect of gaze on PCongE found in previous studies was abolished due to the interfering effect of our emotional stimuli. It is worth noting however, that a post-hoc *t*-test between the direct and averted neutral expression faces also failed to find a significant effect of gaze direction suggesting that, if the presence of emotional expressions did interfere with the effect of gaze on AI, this effect carried over into stimuli that were rated as low in emotional intensity by our validation study (see Supplementary Materials: Table S2).

While neither emotion nor gaze modulated PCongE we did replicate experiment one's finding that raw RTs were modulated by emotional expression. However planned comparisons indicated that the pattern from this experiment was subtly different from that of experiment one with significantly faster RTs for the genuine and polite smiles compared to the neutral face but no significant difference between the frown and neutral condition.

## 4 Experiment 3: does emotional expression modulate compatibility responses to social gestures?

### 4.1 Experiment 3: introduction

The results of both experiment one and experiment two indicated that emotional expression did not significantly modulate the AI of intransitive hand actions. However, this leaves open the possibility that, despite this lack of a strong effect of emotional expression on general, AI emotional expressions can modulate forms of stimulus response compatibility effects (SRCs) that are more social in nature. As has been noted (e.g. Cook, Dickinson, & Heyes, 2012; Farmer et al., 2018; Sartori, Cavallo, Bucchioni, & Castiello, 2012), there are many forms of social action matching in which imitative actions are maladaptive. For example coordinating the coordinated movement of objects (Sacheli, Candidi, Pavone, Tidoni, & Aglioti, 2012), in which one actor releasing the object predicts the other grasping it, or in displays of social dominance in which an expansive posture by one actor leads to a contractive posture in the other (Tiedens & Fragale, 2003).

Handshakes are a particularly overlearned form of complementary action, when someone offers us their right hand to shake, we respond with our own right hand. This anatomical compatibility effect differentiates handshakes from many other forms of interaction, e.g. passing someone an object, or consciously imitating them in which the more natural way for us to interact is with the hand of the opposite laterality but the same spatial location. In support of this studies of AI have tended to find strong spatial compatibility effects with greater congruency effects when responding with the spatially compatible hand compared to the anatomically compatible one (Boyer, Longo, & Bertenthal, 2012; Jiménez et al., 2012). By contrast studies examining handshaking have shown faster responses with the anatomically compatible hand than the spatial compatible one (Flach, Press, Badets, & Heyes, 2010; Liepelt, Prinz, & Brass, 2010). For example, Liepelt et al. (2010) presented participants with images of right and left hands in one of three postures representing three different action affordances and cued them to respond with either the spatially congruent hand (i.e. left hand to right hand image) or the anatomically congruent hand (i.e. right hand to right hand image). The postures displayed were a handshake gesture (communicative), a closed hand (instransitive) or a hand holding an apple (transivitve). The study found that for the transitive and intrasitive gestures participants were faster when responding with their spatially congruent hand, however for the handshake gesture participants responded faster with their anatomically congruent hand.

As well as being an alternative form of SRC from the AI induced by open/closed hand actions studied in experiments one and two, handshakes are also a highly social gesture and are associated with approach behaviour, positive affect and cooperation (Chaplin, Phillips, Brown, Cianton, & Stein, 2000; Dolcos, Sung, Argo, Flor-Henry, & Dolcos, 2012; Schroeder, Risen, Gino, & Norton, 2019). This inherently social nature suggests that the effect of social modulators in modulating hand actions SRC should be enhanced for handshakes. In line with this Liepelt et al. (2010) showed that the SRC effect for handshakes, but not for transitive or intrasitive actions, was modulated by the humaness of the hand. In the case of emotional expressions, one might expect the compatibility effect to be strengthened for genuine and, to a lesser extent, polite smiles but weakened for frowns when compared to neutral. Experiment three sought to test this hypothesis.

### 4.2 Experiment 3: methods

#### 4.2.1 Participants

Thirty participants (15 male) with a mean age of 22.8 (SD = 3.52) took part in the experiment. A sensitivity analysis carried out using G*Power (Faul et al., 2007) indicated that this sample size was sufficient to detect an effect of emotional expression on congruency with an effect size (*f*) of 0.22 with alpha set at 0.05 and beta set at 0.8 (see Supplementary Materials 3.1 for full protocol).

#### 4.2.2 Design and procedure

This experiment assessed the impact of dynamic emotional expressions on participants' tendency to make complementary handshake gestures. The experiment was designed as a 2 (Actor: male, female) × 4 (Emotional Expression: genuine smile, polite smile, neutral, frown) × 2 (Complementarity of Observed and Executed hands: complementary, uncomplementary) within-subjects design. The dependent variables were reaction times and the percentage compatibility effect. The procedure was as in experiment one .

#### 4.2.3 Materials

##### 4.2.3.1 Stimuli preparation

**Fig. 3:**
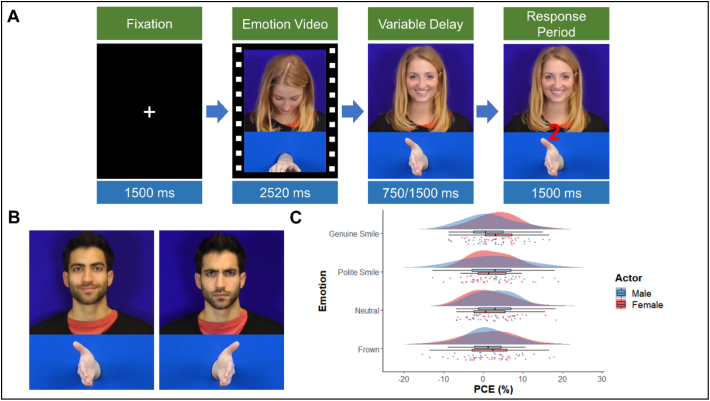
A) Time course of a complementary trial showing the four time periods within each trial. B) Examples of male polite smile left hand and male frown right hand stimuli. C) Raincloud plot showing mean PCompE across emotion and actor. Clouds represent distribution, raindrops represent individual datapoints.

The emotional expression stimuli were taken from the same set of clips as used in experiment one. Handshake stimuli were separately recorded and added to the video clips below the emotional expression clip. For each clip versions were created with either a left or a right hand placed horizontally. Then in the final 14 frames of the video 7 hand images were displayed at a rate of one every two frames so that the hand moved into a vertical position as if offering a handshake. To create baseline trials versions were made in which the hand did not move during those final frames. In addition, the final frame of each video was sampled as a still image and a numerical cue of either “1” (left hand response) or “2” (right hand response) was added to the centre of the clip (see Fig. 3B for example stimuli).

##### 4.2.3.2 Apparatus

The apparatus used to present the stimuli and collect motion data for this study was the same as that used for experiment one. However, rather than attaching the markers to the middle finger and thumb of the right hand, they were attached to the index fingers of the participants right and left hands.

##### 4.2.3.3 Handshake complementarity task

The task consisted of six blocks each of which contained 32 experimental trials plus an initial trial which was discarded during analysis. In addition, 32 baseline trials in which the actor's hand did not move were split among the blocks (Four blocks had five baselines and two blocks had six). Thus, a participant ran through a total of 230 trials, 12 experimental trials per condition. The identity of the actor was counterbalanced across blocks and the order of actor was counterbalanced across participants. The emotional expression, observed action and cued action conditions were randomised across trials in each block. Prior to starting the main task participants completed an additional practice block with a different female actor which consisted of ten trials.

Participants began each trial with their hands laid on the desk in a horizontal position. In each trial a fixation cross was presented at centre for 1500 ms then the video clip of the emotional expression appeared and played while an image of either a left or right hand in a horizontal position was displayed below them. Following this the hand moved into either a handshake position (experimental trials) or remained in the same position (baseline trials). To prevent anticipation effects there was a delay of either 750 ms or 1500 ms before the appearance of the movement cue, this timing was similar to those used in previous studies that probed the impact of social stimuli on automatic imitation (Leighton et al., 2010; Press et al., 2007).. Participants were instructed to move their hand into position “as if you were going to shake someone's hand” as soon as they saw the movement cue. Response times were recorded from the appearance of the cue, and participants had 1500 ms in which to make a response,. The total trial length was either 6270 ms or 7020 ms dependent upon the time delay of the start of the actor's hand movement (see Fig. 3A).

#### 4.2.4 Data processing and analysis

Of the 230 trials for each participant, the first trial from each block along with the 32 baseline trials were removed leaving a total of 192 experimental trials, 12 per condition. Error trials (0.16% of total trials) and trials with RTs less than 50 ms or greater than 1000 ms were excluded from this analysis. In addition, In line with previous studies, (Flach et al., 2010; Liepelt et al., 2010) we categorised trials as either complementary (if the laterality of the observed hand matched the laterality of the executed hand) or uncomplementary (if the laterality of the two hands did not match). For each trial peak velocity for both hands were calculated in the same manner as in experiment one and used to find the reaction time for the cued hand in each trial. These were then used to calculate the mean reaction times which were then used to generate a percentage complementarity effect (PCompE) in the same manner as the PCongE of the previous two studies using the following formula:

$$\mathrm{PCompE}=\frac{\mathrm{MeanUncomplementaryRT}-\mathrm{MeanComplementaryRT}}{\mathrm{MeanOverallRT}}\times 100$$

As in the previous experiments, error trials in which participants did not respond with the cued hand (0% of total trials) along with trials with RTs less than 50 ms or greater than 1000 ms (1.04% of total trials) were excluded from this analysis. No participants had less than 85% valid experimental trials.

### 4.3 Experiment 3: results

Two repeated measures ANOVAs were conducted on the handshake complementarity reaction time data (see Supplementary Materials 3.2 for an analysis of accuracy scores). The first took raw RTs as the DV and actor, emotion and complementarity as the IVs. There was a significant effect of actor, *F*(1, 29) = 11.36, *p =* .002, pƞ^2^ = 0.282, due to faster responses for the female actor (EMM = 498.72, SE = 15.5) compared to male actor (EEM = 507.83, SE = 14.18). A significant effect of emotion was found, *F*(3, 87) = 14.25, *p* < .001, pƞ^2^ = 0.329. Bonferroni corrected pairwise comparisons of the estimate marginal means indicated that this effect was due to significantly slower RTs in the neutral condition (EMM = 510.47, SE = 15.57) than in the genuine smile (EMM = 497.36, SE = 14.45, *p* < .001, *d* = −0.159), polite smile (EMM = 503.79, SE = 14.57, *p* = .018, *d* = −0.081) and frown (EMM = 501.48, SE = 14.78, *p* = .004, *d* = −0.108) conditions. RTs for the polite smile were also significantly slower than for the genuine smile (p = .004, *d* = −0.081). No other significant differences between emotional expressions were found. A significant effect of complementarity was also found, *F*(1, 29) = 10.18, *p* = .003, pƞ^2^ = 0.26, due to participants being faster to respond in the complementary condition (EMM = 497.4, SE = 14.19) compared to the uncomplementary condition (EEM = 509.15, SE = 15.56). There was no significant interaction between actor and emotion *F*(3, 87) = 0.18, *p* = .911, pƞ^2^ = 0.006; between actor and complementarity, *F*(1, 29) = 0.10, *p* = .758, pƞ^2^ = 0.003; or between emotion and complementarity, *F*(3, 87) = 0.53, *p* = .665, pƞ^2^ = 0.018. The three-way interaction was also non-significant, *F*(3, 87) = 0.98, *p* = .408, pƞ^2^ = 0.033 (see Table 3).

**Table 3:** Means and standard deviations for complementary, uncomplementary and percentage complementarity effects for each condition in experiment three.

Conditions		Comp (ms)		Incomp (ms)		PCompE (%)	
Actor	Emotion	Mean	SD	Mean	SD	Mean	SD
Female	Genuine Smile	482.81	78.84	501.04	85.57	3.55	5.49
	Polite Smile	493.49	91.73	505.06	86.62	2.48	5.32
	Neutral	500.52	77.06	510.62	95.66	1.57	5.41
	Frown	493.38	83.28	502.79	93.77	1.58	6.70
Male	Genuine Smile	498.89	79.18	506.70	80.43	1.55	6.07
	Polite Smile	501.23	67.23	515.37	83.36	2.35	7.46
	Neutral	507.22	80.64	523.52	93.68	2.97	5.39
	Frown	501.68	82.66	508.05	77.77	1.35	6.52

The second ANOVA took PCompEs as the DV and actor and emotion as the IVs. This analysis found no significant effect of either actor, *F*(1, 29) = 0.11, *p =* .739, pƞ^2^ = 0.004; or emotion, *F*(3, 87) = 0.5, *p =* .682, pƞ^2^ = 0.017. Nor was there a significant interaction between the two IVs, *F*(3, 87) = 1.05, *p* = .374, pƞ^2^ = 0.035 (see Fig. 3C and Table 3).

### 4.4 Experiment 3: discussion

Despite the use of a more social stimuli which had a distinct SRC profile to the actions used in the previous studies the results of experiment three were consistent with those of experiments one and two. As in those studies there was a significant main effect of emotion on raw RTs which in this study appeared to be more supportive of the arousal explanation. We also replicated previous findings of a significant complementarity effect with participants responding faster to a complementary compared to non-complementary hand (Flach et al., 2010; Liepelt et al., 2010). However, there was no effect of either actor or emotional expression on PCompEs and neither was there an interaction between these two factors and complementarity in the raw RT data. Moreover, there was also no significant main effects or interactions when we distinguished between handshakes made with the right hand, which is the hand used for social handshakes, and those made with the left hand, which are less overlearned and less socially meaningful (see Supplementary Materials 3.3 for details of this analysis). This suggests that the effect of handshake complementarity is not strongly modulated by the amount of experiences with or social meaning of the hand used.

## 5 Experiment 4: does emotional expression modulate imitative response to affective social gestures?

### 5.1 Experiment 4: introduction

Since our first three studies failed to find any significant effect of emotions on SRCs, even when the gesture used had a clear social meaning, in experiment four we sought to investigate the effect of dynamic emotional expressions when participants had to respond to social gestures which have clear affective meanings.

This study was motived by recent results reported by Cracco, Genschow, Radkova, and Brass (2018) who investigated the AI of gestures with an inherently pro-social (thumbs up) or anti-social (middle finger) meaning. They found that participants showed greater AI of the pro-social compared to anti-social gesture and that the difference between the two conditions was greater if the participants were primed with a pro-social as opposed to an anti-social context. These results are in line with theories suggesting imitation is deployed strategically to encourage affiliation (Chartrand & Lakin, 2013; Wang, & Hamilton, A. F. de C., 2012).

Our fourth experiment can be considered conceptually similar to experiments two and three from Cracco, Genschow, et al. (2018) except that while they primed participants with a pro- or anti-social context using a scrambled sentence task the current experiment sought to prime participants by using emotional expressions. Because this experiment was more strongly focused on the specific valence of the emotions than the previous ones, we chose to simplify our design by removing the neutral and polite smile conditions and focusing on the difference between the genuine smile and the frown expressions.

### 5.2 Experiment 4: methods

#### 5.2.1 Participants

49 participants (25 male) took part in the experiment. Six male participants were excluded from the final analysis as they as they had less than 85% valid RTs in their non-baseline trials leaving a final sample of 43 participants with a mean age of 26.79 (SD = 7.76). A sensitivity analysis carried out using G*Power (Faul et al., 2007) indicated that this sample size was sufficient to detect an effect of emotional expression on congruency with an effect size (*f)* of 0.18 with alpha set at 0.05 and beta set at 0.8 (see Supplementary Materials 4.1 for full protocol).

#### 5.2.2 Design and procedure

This experiment assessed the impact of dynamic emotional expressions on participants' tendency to make compatible handshake gestures. The experiment was designed as a 2 (Type of Gesture: thumbs up, middle finger) × 2 (Emotional Expression: genuine smile, frown) × 2 (Congruency of Observed and Executed Actions: congruent, incongruent) within-subjects design. The dependent variables were reaction times and the percentage compatibility effect. The procedure was as in experiment one .

#### 5.2.3 Materials

##### 5.2.3.1 Stimuli preparation

The emotional expression stimuli were taken from the same set of clips as used in experiment one. Since there were only two emotional expression in this study the diversity of stimuli was increased by using two different actors for each of the two expressions. Only female actors were used as the female stimuli had the highest ratings on the appropriate expressions, including high ratings for genuineness of smile and because no effects of actor gender on congruency had been seen in previous experiments. The actors chosen for the genuine smile stimuli were highest rated for positivity of their smile, while those chosen for the frowns were lowest rated for positivity of their frown expression.

**Fig. 4:**
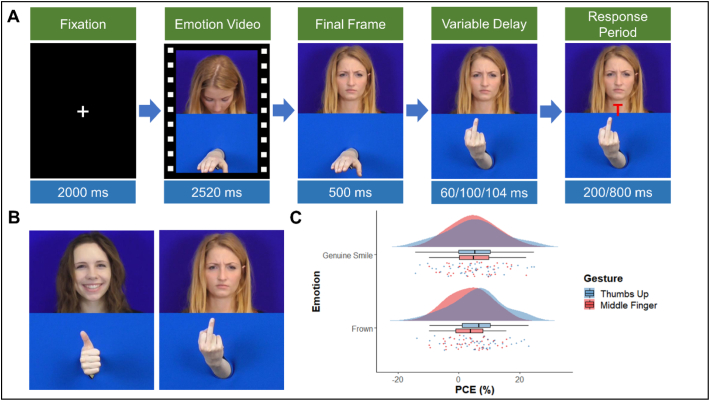
A) Time course of an incongruent trial showing the four time periods within each trial. B) Examples of genuine smile thumbs up and frown middle finger stimuli. C) Raincloud plot showing mean PCongE across emotion and actor. Clouds represent distribution, raindrops represent individual datapoints.

Gesture stimuli were separately recorded and added to the video clips below the emotional expression clip. A baseline stimuli of a hand in a horizontal open position was placed below the face in each frame of the video, then the final frame of the video was taken as a still image and two additional images were created. The first of these showed the hand in its final position (thumbs up or middle finger) and the second added a cue of either “T” (thumbs up response) or “M” (middle finger response) to the centre of the clip (see Fig. 4B).

##### 5.2.3.2 Apparatus

The apparatus used to present the stimuli and collect motion data for this study was the same as that used for experiments one and two except that the markers were attached to the thumb and middle finger of the participants dominant hand and Psychtoolbox (Kleiner et al., 2007) rather than MATLAB was used to display stimuli.

##### 5.2.3.3 Automatic imitation task

The task consisted of eight blocks each of which contained 24 experimental trials. In addition, 48 baseline trials in which the actor's hand did not move were split equally among the blocks. During presentation a break was added in the middle of each block to allow participants to rest their hand and an extra trial was added at the start of each block and after each break which were discarded during analysis. Thus, each block consisted of 32 trials and in total participants completed 230 trials. The emotion shown was counterbalanced across blocks and the order the of emotions was counterbalanced across participants. The actor, observed action and cued action conditions were randomised across trials in each block. Prior to starting the main task participants completed an additional practice block which consisted of ten trials with the opposite emotion and actors to that seen in the first experimental block.

The timings of each trial was based on those used by Cracco, Genschow, et al. (2018). Participants began each trial with their hands raised from the desk in a horizontal position. In each trial a fixation cross was presented at centre for 2000 ms then the video clip of the emotional expression appeared and played. Following this the final frame of the video remained on the screen for 500 ms before the actor's hand moved into either a gesture position (experimental trials) or remained in the same position (baseline trials). To prevent anticipation effects there was then a delay of either 60 ms, 100 ms or 140 ms before the appearance of the response cue. Response times were recorded from the appearance of the cue, and participants had 1500 ms in which to make a response. The total trial length was either 7080 ms or 7120 or 7160 ms dependent upon the time delay of the cue appearance (see Fig. 4A).

#### 5.2.4 Data processing and analysis

Of the 256 trials for each participant, the first and 17th trials from each block along with the 48 baseline trials were removed leaving a total of 192 experimental trials, 24 per experimental condition. Peak velocity, reaction time and PCongE were calculated for each condition in the same manner as in experiment one. Trials with RTs less than 50 ms or greater than 1000 ms were excluded from this analysis (5.88% of total trials). Due to the difficulty of identifying error trials from the motion data in this experiment we did not remove error trials from the analysis. Participants who had less than 85% valid experimental trials were not included in the final analysis (six participants).

### 5.3 Experiment 4: results

Two repeated measures ANOVAs were conducted on the AI data. The first took raw RTs as the DV and emotion, observed gesture and congruency as the IVs. Responses were faster for the genuine smile (EMM = 577.56, SE = 13.76) compared to the frown (EEM = 585.07, SE = 14.11) videos, however this did not reach significance, *F*(1, 42) = 3.84, *p =* .057, pƞ^2^ = 0.084. No significant effect of observed gesture was found, *F*(1, 42) = 0.94, *p* = .338, pƞ^2^ = 0.022. However, a significant effect of congruency was found, *F*(1, 48) = 53.24, *p* < .001, pƞ^2^ = 0.559, due to participants being faster to respond in the congruent condition (EMM = 566.45, SE = 13.11) compared to the incongruent condition (EEM = 596.18, SE = 14.75). There was no significant interaction between emotion and gesture *F*(1, 42) = 0.08, *p* = .775, pƞ^2^ = 0.002; between emotion and congruency, *F*(1, 42) = 0.06, *p* = .802, pƞ^2^ = 0.002; or between gesture and congruency, *F*(1, 42) = 0.91, *p* = .346, pƞ^2^ = 0.021. The three-way interaction was also non-significant, *F*(1, 42) = 1.74, *p* = .195, pƞ^2^ = 0.040 (see Table 4).

**Table 4:** Means and standard deviations for congruent, incongruent and percentage congruency effects for each condition in experiment four.

Conditions		Cong (ms)		Incong (ms)		PCongE (%)	
Gesture	Emotion	Mean	SD	Mean	SD	Mean	SD
Thumbs Up	Genuine Smile	559.66	88.04	591.20	97.59	5.11	9.35
	Frown	565.30	87.46	602.19	103.66	6.04	8.36
Middle Finger	Genuine Smile	565.12	92.99	594.24	100.93	4.99	7.24
	Frown	575.73	94.29	597.06	97.68	3.60	6.60

The second ANOVA took PCongEs as the DV and emotion and observed gesture as the IVs. This analysis found no significant effect of either emotion, *F*(1, 42) = 0.09, *p =* .772, pƞ^2^ = 0.002; or observed gesture, *F*(1, 42) = 0.621, *p =* .435, pƞ^2^ = 0.015. Nor was there a significant interaction between the two IVs, *F*(1, 42) = 1.71, *p* = .199, pƞ^2^ = 0.039 (see Fig. 4C and Table 4).

### 5.4 Experiment 4: discussion

Experiment four investigated the effect of emotional expressions on the AI of meaningful gestures. Consistent with the findings of the previous experiments, we found a significant effect of congruency in the mean RTs but did not find any significant effect of the type of emotional expression observed on PCongEs. Moreover, we failed to find any evidence of an interaction between emotional expression and observed gesture on PCongEs even though in this experiment there was a clear congruency relationship between the observed expressions and gestures (genuine smile and thumbs ups vs. frown and middle finger). In addition the effect of emotional expression on overall reaction times did not reach significance, which supports the arousal interpretation of our previous studies effects given that the difference in ratings of intensity between the genuine smile and the frown expressions was much smaller than the difference in their ratings of positivity.

As well as finding no effect of emotional faces we also failed to replicate the findings of Cracco, Genschow, et al. (2018) as we did not find any difference in PCongEs between the pro and anti-social gestures or an interaction between gesture and congruency in the raw RTs. One possible explanation for this failure to replicate is that the effect of gesture was counteracted by the presence of our emotional faces however this seems dubious given that Cracco and colleagues found that the use pro and anti-social primes did not abolish the basic effect of the gestures on congruency. A possible alternative reason for the failure to replicate the earlier finding relates to the nature of the gesture stimuli used. While in our experiment the thumbs up and middle finger stimuli were directed towards the participant as if they were being made by the actors, in Cracco and colleagues' study the stimuli were presented as directed away from the participant towards an unseen other person. This may have influenced participants' perception of the observed gesture's relationship to their executed gesture with Cracco and colleague's stimuli being interpreted as a joint evaluation of an unseen stimuli/person while our stimuli were interpreted as an evaluation of the participant themselves. Further research is necessary to fully understand how the orientation of social gestures interacts with AI. An addition difference between the two studies is that participants in our study only responded with their dominant hand, Cracco and colleagues had their participants use one hand for each gesture which may have allowed for a more automatic response to the stimuli.

## 6 Experiment 5: do static and dynamic emotional expressions differentially modulate automatic imitation?

### 6.1 Experiment 5: introduction

The results of our first four experiments suggest that dynamic facial expressions do not have a moderating effect on the AI of hand actions even when those hand actions are distinctly social (experiments three and four) or have a clear affective valence themselves (experiment four). This pattern of null results appears to raise questions about the replicability of the previously reported effects of emotional expressions on AI (Butler et al., 2016; Rauchbauer et al., 2015; Rauchbauer et al., 2016) particularly since the meta-analysis conducted by Butler and colleagues suggested that the effect of emotion only robustly occurred when contrasting the effects of happy expressions to neutral expressions.

However, there are several discrepancies between the experiments in this paper and those reported previously. Most notably we employed dynamic emotional expressions while the previous studies used static images all taken from the same NimStim database of facial emotions (Tottenham et al., 2009). While previous studies have suggested that in general dynamic emotional stimuli should be expected to lead to enhanced effects (Krumhuber et al., 2013) it is possible that this does not apply to AI. Another possibility is that while the expressions in the NimStim database are rather exaggerated, our dynamic stimuli were designed to be naturalistic with the actors being instructed to make expressions in the context of seeing someone at a social gathering. While the dynamic and naturalistic nature of our stimuli mean that they have higher ecological validity than do the static faces used in previous studies it is possible that they were also less attentionally salient and so did not have a powerful enough effect on participants imitative responses.

As well as these differences in the nature of the emotional stimuli there are other differences between our experiments and previous studies that could potentially explain the difference in findings. While our experiments used a range of hand actions it is notable that all of them involved the whole hand whereas the previous studies all used the finger lifting task developed by Brass et al. (2000). In addition, the experiments reported here used a mixed of pre-specified responses and cued response while the previous studies all employed cued responses. In order to test whether these differences in stimuli or procedure could account for the differences in findings between our studies and those that had previously found that emotional expression modulated AI. We conducted a final experiment which sought to directly compare the effect of our dynamic stimuli with the static stimuli used in previous task while keeping the procedure as close to that laid out by Butler et al. (2016)’s second experiment as possible.

### 6.2 Experiment 5: methods

#### 6.2.1 Participants

35 participants (16 male) with a mean age of 25.91 (SD = 6.86) took part in the experiment. A sensitivity analysis carried out using G*Power (Faul et al., 2007) indicated that this sample size was sufficient to detect an effect of emotional expression on congruency with an effect size (*f)* of 0.22 with alpha set at 0.05 and beta set at 0.8 (see Supplementary Materials 5.1 for full protocol).

#### 6.2.2 Design and procedure

This experiment assessed the impact of dynamic and static positive and negative emotional expressions on participants' tendency to imitate positive and negative social gestures. The experiment was designed as a 2 (Stimuli Type: dynamic video, static image) × 3 (Emotional Expression: genuine smile, neutral, frown) × 2 (Congruency of Observed and Executed Actions: congruent, incongruent) within-subjects design. The actions were the raising of the middle or index finger of the right hand. The dependent variables were reaction times and percentage congruency effect. In addition, participants in this task were asked to rate the actor's emotional expressions and facial characteristics for both the static and dynamic stimuli (see Supplementary Materials 5.2 for full details and results). Participants first completed the AI task then they did the ratings task.

#### 6.2.3 Materials

##### 6.2.3.1 Stimuli preparation

The dynamic emotional expression stimuli were taken from the same set of clips used in the previous studies. The static emotional expression stimuli were taken from the NimStim database of expressions (Tottenham et al., 2009). The expressions from all eight of the dynamic stimuli models (four male and four female) were used in the study and eight (four male and four female) white models (to match to the ethnicity of our dynamic stimuli models) were selected from the NimStim database.

**Fig. 5:**
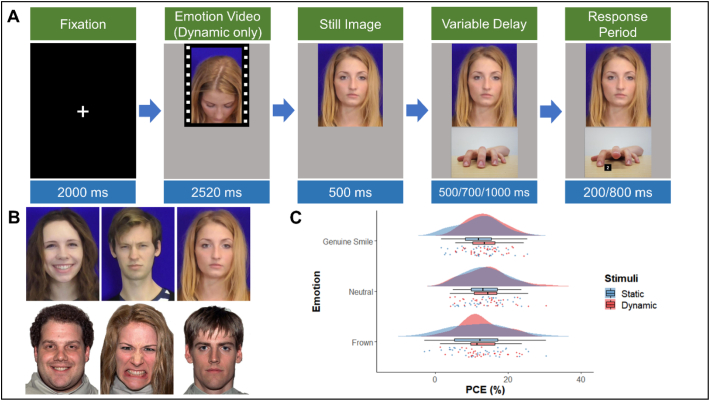
A) Time course of a congruent trial showing the five/four time periods within each trial. B) Examples of smile, frown and neutral, dynamic (top) and static (bottom) stimuli. C) Raincloud plot showing mean PCongE across emotion and stimuli type. Clouds represent distribution, raindrops represent individual datapoints.

The finger lifting stimuli were the same used in Butler et al. (2016). In each trial the video/image was displayed in the top half of the screen while the finger lifting stimuli were displayed in the bottom portion of the screen (see Fig. 5B).

##### 6.2.3.2 Apparatus

The experiment was run in MATLAB using Psychtoolbox (Kleiner et al., 2007) to display the stimuli and collect response data. Unlike previous studies participants viewed stimuli on a 22-inch computer monitor with a 4:3 aspect ratio. Participants responded using a standard computer keyboard.

##### 6.2.3.3 Automatic imitation task

The task consisted of four blocks each of which contained 96 experimental trials. During presentation a break was added in the middle of each block to allow participants to rest. The type of stimuli shown alternated across blocks and the order that the stimuli types were shown in was counterbalanced across participants. The emotion, observed action and cued action conditions were randomised across trials in each block. Prior to starting the main task participants completed an additional 10 trial practice block with the opposite stimuli type to that seen in the first experimental block.

Participants began each trial with the index finger of their right hand on the “<” key and the middle finger of their right hand on the “>” key on the keyboard. In each trial a fixation cross was presented at centre for 500 ms. For the dynamic stimuli the video of the emotional expression appeared and was played through and the final frame was displayed for 500 ms. For the static stimuli the image was displayed for 500 ms. Following this the hand stimuli in the start position (index and middle fingers flat on the desk appeared. To prevent anticipation effects there was then a delay of either 500 ms, 700 ms or 1000 ms, these timing were based on those used by Butler et al. (2016).After this delay the hand shifted to show either the index or middle finger lifted and at the same time a movement cue of either “1” (index finger) or “2” middle finger appeared. Participants then had up to 2000 ms time to respond to the cue. Following their response, the fixation screen was displayed again, and participants had to press down on both keys at which point the next trial began. Thus the total trial length for dynamic stimuli was up to either 6020 ms or 5220 or 7520 ms dependent upon the time delay of the cue appearance and the time taken to respond while the total trial length for the static stimuli was up to either 3500 ms or 3700 or 4000 ms dependent upon the time delay of the cue appearance and the time taken to respond (see Fig. 5A).

#### 6.2.4 Data processing and analysis

In line with Butler et al. (2016) in this experiment we used all 384 trials, 32 per experimental condition, in our analysis. Reaction time and PCongE were calculated for each condition. As in the previous studies, error trials in which the participant made the incorrect movement (4.15% of total trials) and trials with RTs less than 50 ms or greater than 1000 ms (0.55% of total trials) were excluded from this analysis. No participants had less than 85% valid experimental trials.

### 6.3 Experiment 5: results

Two repeated measures ANOVAs were conducted on the data (see Supplementary Materials 5.3 for an analysis of accuracy scores). The first took raw RTs as the DV and stimuli type, emotion and congruency as the IVs. There was a significant effect of stimuli type, *F*(1, 34) = 8.23, *p =* .007, pƞ^2^ = 0.195, due to faster responses for the static (EMM = 449.37, SE = 8.87) compared to dynamic (EMM = 463.21, SE = 11.21) stimuli. There was no significant effect of emotion, *F*(2, 68) = 1.51, *p =* .228, pƞ^2^ = 0.043. However, a significant effect of congruency was also found, *F*(1, 34) = 233.71, *p* < .001, pƞ^2^ = 0.873, due to participants being faster to respond in the congruent condition (EMM = 426.42, SE = 8.83) compared to the incongruent condition (EEM = 486.16, SE = 11.06). There was no significant interaction between stimuli type and emotion, *F*(2, 68) = 0.34, *p* = .710, pƞ^2^ = 0.010; between stimuli type and congruency, *F*(1, 34) = 1.42, *p* = .242, pƞ^2^ = 0.040; or between emotion and congruency, *F*(2, 68) = 1.67, *p* = .197, pƞ^2^ = 0.047. The three-way interaction was also non-significant, *F*(2, 68) = 0.27, *p* = .763, pƞ^2^ = 0.008 (see Table 5).

**Table 5:** Means and standard deviations for congruent, incongruent and percentage congruency effects for each condition in experiment five.

Conditions		Cong (ms)		Incong (ms)		PCongE (%)	
Stimuli type	Emotion	Mean	SD	Mean	SD	Mean	SD
Video	Genuine Smile	429.81	59.43	492.40	73.35	13.57	4.66
	Neutral	433.48	63.30	497.80	77.81	13.94	6.12
	Frown	433.90	61.69	491.87	72.69	12.59	5.63
Still	Genuine Smile	419.74	46.80	475.81	62.45	12.06	5.65
	Neutral	419.22	50.20	481.17	62.89	13.48	5.16
	Frown	422.35	47.87	477.93	60.35	12.09	7.83

The second ANOVA took PCongEs as the DV and emotion and observed gesture as the IVs. This analysis found no significant effect of either stimuli type, *F*(1, 34) = 1.51, *p =* .227, pƞ^2^ = 0.043; or emotion, *F*(2, 68) = 1.62, *p =* .207, pƞ^2^ = 0.045. Nor was there a significant interaction between the two IVs, *F*(2, 68) = 0.35, *p* = .708, pƞ^2^ = 0.010 (see Fig. 5C and Table 5).

### 6.4 Experiment 5: discussion

The final experiment of our paper sought to directly compare the effect of viewing static and dynamic emotional expressions using the same experimental procedure employed by Butler et al. (2016) in their second experiment. This design allowed us to control for the possibility that the null results found in our previous studies were due to one or more of a number of factors including: the naturalistic stimuli we used; the dynamic nature of our stimuli; the type of hand actions; or some other factor in our experimental designs.

The results of experiment five essentially replicated those of our previous studies with a significant effect of congruency in the raw RTs indicating that, once again, we elicited a congruency effect. However as with all our previous studies examination of the PCongEs we found no significant effect of emotion, suggesting that emotional expressions did not modulate AI. Moreover, we found no effect of stimuli type on PCongEs nor any interaction between emotion and stimuli type. This indicates that the static NimStim faces used in previous studies were no more effective in modulating AI than were our dynamic stimuli. It is worth noting that there was a main effect of stimuli type on RTs which could potentially be an arousal related effect given that participants in this study rated the static stimuli as more intense that the dynamic stimuli. However, in this study there was no effect of emotion on raw RTs.

## 7 Meta-analysis and Bayesian *t*-tests

### 7.1 Meta-analysis

To examine the effect of emotional expressions on SCRs across all five of our studies we conducted series of random effects meta-analyses in R using the *meta* package (Balduzzi, Rücker, & Schwarzer, 2019; Schwarzer, 2020). We followed Butler et al. (2016) in running analyses comparing the effect of Genuine Smile vs Frown, Genuine Smile vs Neutral and Frown vs Neutral on our percentage congruency/compatibility effect and separately on the raw RTs for both congruent/compatible and incongruent/compatible trials. As experiment four did not include a neutral expression condition data from that study was only used in the Genuine smile vs Frown comparisons. Since none of our additional factors (actor, gaze direction, observed gesture or stimuli type) showed an effect on congruency/compatibility we collapsed our data across these factors.

**Fig. 6:**
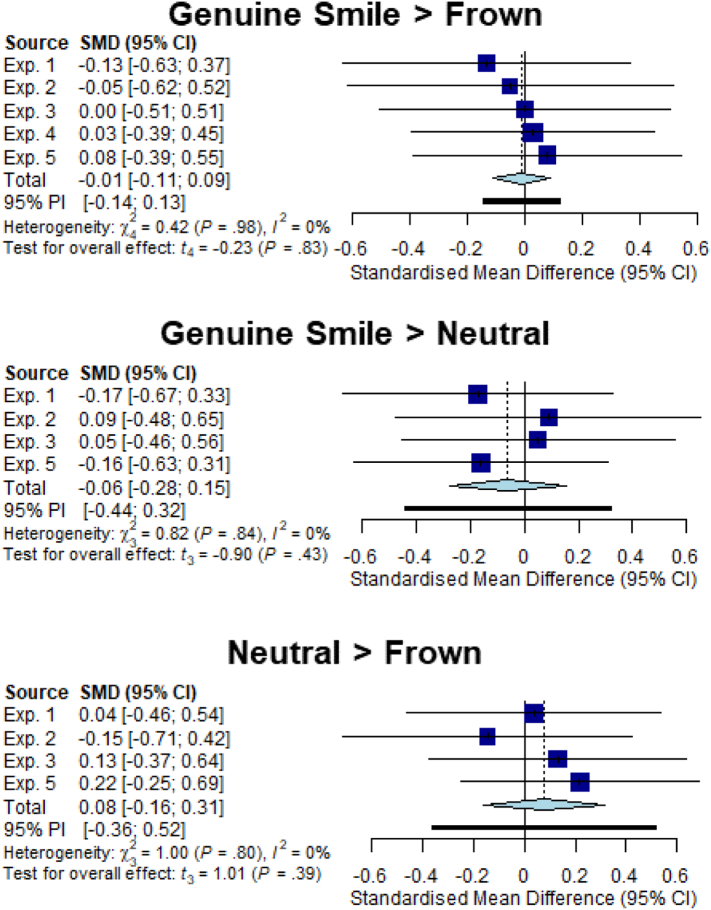
Forest plots of the meta-analyses on the percentage congruency/compatibility effects for the comparisons between the genuine smile, frown and neutral conditions.

Fig. 6 shows the results of our meta-analysis for the percentage congruency/compatibility effects. As can be seen there was no significant increase in PCong/CompEs for the Genuine Smile compared to the Frown, *t*(4) = −0.23, *p* = .83. Nor was there a significant increase in PCong/CompEs for the Genuine Smile compared to Neutral expression, *t*(3) = −0.90, *p* = .43, or for the Neutral expression compared to the Frown, *t*(3) = −1.01, *p* = .39. We did find evidence for significantly faster responses to the Genuine Smile compared to the Frown condition in congruent trials, *t*(4) = −3.69, *p* = .02. However, there was no significant evidence for a difference in congruent trial reaction times between the Genuine Smile and Neutral expressions, *t*(3) = −2.66, *p* = .08, or the Frown and Neutral expressions, *t*(3) = −1.75, *p* = .18. These findings contrast with those of Butler et al. whose own meta-analysis did not find a difference in congruent trial RTs between Happy and Angry faces but did find faster RTs in their Angry compared to Neutral congruent trials. For the incongruent trials all three contrasts showed significant effects (Genuine Smile vs Frown: *t*(3) = −3.37, *p* = .03, Genuine Smile vs Neutral: *t*(3) = −4.90, *p* = .02, Neutral vs Frown: *t*(3) = 5.85, *p* = .010). However, as with the congruent trials, these effects tended to be the reverse of those seen in Butler et al.'s analysis. For example, Butler et al. found evidence for slower RTs in the Happy incongruent trials compared to the Angry and Neutral ones while we found evidence for faster RTs for incongruent Genuine Smile trials compared to incongruent Frown and Neutral trials .

### 7.2 Bayesian *t*-tests

In addition to the frequentist meta-analysis we also followed previous studies (Butler et al., 2016; Farmer et al., 2016) in running a series of Bayesian *t*-tests on our data. In contrast to frequentist hypothesis tests, which can only indicate if a specified hypothesis was significant, Bayesian hypothesis testing involves the calculation of Bayes Factors (BFs), which indicate the relative strength of evidence for one hypothesis over another (Dienes, 2014).

In our case, we sought to find the BF01, i.e. the odds of favouring the null hypothesis over the alternative, in order to assess whether our findings gave good evidence to accept the null hypothesis (and consequently, accept that there is no effect of emotional expression on SRC effects). BFs can range from zero to an infinite value, whereby a value of one does not favour either theory, and values above 1 indicate increasing evidence for one alternative over the other. Jeffreys (1961) suggests that odds greater than 3 should be considered as some evidence in favour of one hypothesis over another, whereas odds greater than 10 should be regarded as strong evidence.

We used JASP (JASP Team, 2019) to carry out one tailed t-tests using both Frequentist and Bayesian methods. Since we originally hypothesised that more positive expressions would lead to greater automatic imitation, we ran a series of three different contrasts. The first set examined whether the PCon\CompEs were greater for Genuine Smile trials than Frown trials., The second set examined whether PCong\CompEs were greater for Genuine Smile trials compared to Neutral trials and the third examined whether PCong\CompEs were greater for Neutral trials compared to Frown trials. The first set was run for all five experiments, while the other two sets were run for all experiments apart from experiment four. To test the robustness of our Bayesian analysis we conducted two Bayesian t-tests for each set of contrasts, one using an informed prior distribution and the other using a default prior distribution. The default prior used a central Cauchy distribution with a scale parameter of r = √ 2/2, as suggested by Rouder, Speckman, Sun, Morey, and Iverson (2009). The informed prior used a t − distribution with a location parameter of μ = 0.35 a spread of *r* = 0.102 and three degrees of freedom. This distribution was derived from Gronau, Ly, and Wagenmakers (2020) and has been suggested to be a typical informed prior in the field of psychology (Stefan, Gronau, Schönbrodt, & Wagenmakers, 2019).

Table 6 shows the results of the analyses. As can be seen none of the frequentists t-tests showed significant support for the alternative hypothesis. For the Bayesian t-tests the results were generally in favour of the null hypothesis. For the Genuine Smile > Frown contrasts when using both informed and uniformed priors there was moderate to strong support for the null in experiments one, two and four and anecdotal support for the null hypothesis in experiment five. For experiment three, there was anecdotal support for the alternative hypothesis when using the informed prior but anecdotal support for the null hypothesis when using the uninformed prior. For the Genuine Smile > Neutral contrasts there was strong or moderate support for the null when using both the informed and uninformed prior in experiments one, three and five, while in experiment two there was moderate support for the null when using the uninformed prior and anecdotal support for the null when using the informed prior. Finally for the Neutral > Frown contrast there was moderate support for the null when using either prior in experiments one and two but experiments three and five showed anecdotal support for the alternative hypothesis when using the informed prior and anecdotal support for the null when using the uninformed prior.

**Table 6:** Results of Frequentist and Bayesian one tailed t-tests comparing PCong\CompEs effects for Genuine Smile (GSm), Frown (Frn) and Neutral (Ntl) conditions across studies.

Exp	Contrast	Frequentist			BF_01_ Informed	BF_01_ Uninformed
		t-Value	*p*-Value	Effect Size (d)		
1	GSm > Frn	−0.87	0.804	−0.16	12.75^b^	9.01^a^
	GSm > Ntl	−1.03	0.313	−0.18	14.84^b^	9.74^a^
	Ntl > Frn	0.21	0.416	0.04	3.41^a^	4.40^a^
2	GSm > Frn	−0.24	0.592	−0.05	4.75^a^	5.51^a^
	GSm > Ntl	0.43	0.336	0.09	1.98	3.26^a^
	Ntl > Frn	−0.79	0.781	−0.16	8.79^a^	7.68^a^
3	GSm > Frn	1.20	0.119	0.22	0.78	1.53
	GSm > Ntl	0.28	0.392	0.05	3.21^a^	4.11^a^
	Ntl > Frn	−1.06	0.850	−0.19	0.99	1.84
4	GSm > Frn	0.29	0.386	0.05	4.33^a^	4.76^a^
5	GSm > Frn	0.71	0.241	0.12	1.79	2.90
	GSm > Ntl	−1.22	0.884	−0.21	20.31^b^	11.27^b^
	Ntl > Frn	1.53	0.068	0.26	0.44	1.04

a = moderate evidence for the null.

b = strong evidence for the null.

## 8 General discussion

### 8.1 Accepting the null

The five studies presented in this paper all investigated the relationship between emotional expression and various forms of stimulus response compatibility (SRC) mostly focusing on the automatic imitation (AI) of hand actions (Experiments one, two and four) but also looking at the AI of finger movements (Experiment five) and complementary responses to handshakes (Experiment three). In all these studies we examined the same key question, do observed emotional expressions modulate SRC. Both the analyses of individual studies and the overall meta-analysis found no evidence to support a modulating effect of emotional expressions on SRC. These findings are strikingly consistent despite the studies involving a range of different motor actions and having a number of other differences in their design such as the use of cued vs pre-specified movements or differences in the delay between the observation of emotional expression, action stimuli and required response. In addition, the social meaning of the observed gestures does not seem to have increased any modulating effect of the emotional expressions as observing either handshakes or gestures with their own affective meaning also failed to show any effect of emotional expression.

These results help to add some clarity into a research area which to date has thrown up conflicting findings. First, it is worth noting that our findings are in agreement with those of Crescentini et al. (2011) and Grecucci et al. (2013) both of which also found no evidence of an effect of negative emotional faces on AI. They are also partially in line with the results of the two studies from of Butler et al. (2016) which also failed to find a significant interaction (although a Bayesian analysis did find moderate support in favour of differences in congruency effects between Happy and Angry faces in experiment one and between Angry and Neutral faces in experiment two). Considering the literature as a whole it is notable that the only study to report a strong effect of emotional faces on AI was Rauchbauer et al. (2015), who found evidence that participants responded faster to Happy faces than Angry faces from both a racial ingroup and a racial outgroup. However, even this finding was not directly replicated in a second study (Rauchbauer et al., 2016) which instead found an interaction between group and emotion with greater AI for Happy compared to Angry faces for the ingroup and greater AI for Angry compared to Happy faces for the outgroup. Based on the available evidence it therefore seems likely that observed emotional expression have, at most, a minimal modulating effect on imitative response.

The lack of strong evidence for the modulating effect of emotional expression on automatic imitation contributes to a wider literature regarding the influence of top-down factors on automatic imitation. As discussed in the introduction a number of theories have claimed that imitative behaviour has an affiliative function that serves to bind together social groups (Chartrand & Bargh, 1999; Lakin et al., 2003; Wang & de C Hamilton, 2014). One form of evidence for these theories were findings that factors such as gaze (Wang & de C Hamilton, 2014; Wang, Newport, & Hamilton, 2011) and pro-social priming (Leighton et al., 2010; Wang & de C Hamilton, 2013) modulated AI. In contrast a number of studies have found that AI is not sensitive to social factors including status (Farmer et al., 2016) and animacy cues (Cracco, Bardi, et al., 2018), while another recent study failed to replicate the effect of pro-social priming on AI (Newey, Koldewyn, & Ramsey, 2019). In addition to these findings other studies have suggested that performance in AI does not correlate with traits related to social cognition including empathy and autism (Butler et al., 2015; Cracco, Bardi, et al., 2018) or with pro-social behaviour (Galang & Obhi, 2020). These studies have lead to debate as to the relationship between AI and social cognition (Cracco & Brass, 2019; Ramsey, 2018). To the extent that the modulation of AI by social factors can be viewed as evidence for a specifically social function (Farmer et al., 2018), our findings add to this debate.

### 8.2 Limitations

While the studies reported here all came to largely similar conclusions there are some limitations to how far these conclusions can be applied more generally. First and most importantly our studies had relatively low power to detect our key effects of interest, i.e. the effect of emotional expression on congruency. All our studies had sufficient power to detect an effect size of *d* = 0.5 which Cohen (1988) interprets as a medium effect size, however they lacked sufficient power to detect smaller effect sizes particularly those below *d* = 0.4. It is not entirely clear how great an effect to expect emotional expressions to exert upon automatic imitation. Rauchbauer et al. (2015) found an effect of *d* = 0.69 when comparing congruency effects when viewing smiles vs frowns, while a recent review of the effect of self-other focus on automatic imitation found an average effect size of *d* = 0.58 (Genschow, Schuler, Cracco, Brass, & Wänke, 2019). On the other hand more recent studies (Butler et al., 2016; Rauchbauer et al., 2016) have found that the average effect size of emotional expressions on automatic imitation is close 0.3 which is also closer to an estimate of the generic effect of social priming on behaviour and the default informed prior we used in our Bayesian analyses. If the true effect of emotional expression on automatic imitation is closer to this size it is possible that our studies simply did not have sufficient power to detect it.

We have attempted to address this limitation by running a meta-analysis on the effects for all five experiments. This failed to find evidence in support of the alternative hypotheses that emotional expressions would show significant differences from neutral expressions or from each other. Moreover, our Bayesian analysis, which is not dependent on a set sample size to estimate the extent to which the null is supported (Rouder, 2014; Wagenmakers, 2007), found consistent strong to moderate evidence for the null using both informed and uninformed priors in 8 out of the 13 contrasts we tested and no contrast where both priors indicated even anecdotal support for the alternative hypothesis. Overall, we therefore believe that the evidence across studies clearly points towards the null even if the low power means that we cannot conclusively exclude a small effect of emotional expression on automatic imitation.

A second limitation to our study relates to the question of how far our stimuli was perceived as naturalistic by participants. While our use of dynamic facial expressions was more naturalistic than the static emotional expressions used in previous studies it should be noted that the combination of hand gestures with the face stimuli was not done in an anatomically plausible manner in any of our experiments. Due to this it is unclear the extent to which our participants perceived the hand movements they observed as being carried out by the person whose face they saw. However, previous studies that have found evidence in support of effects of gaze (Wang & de C Hamilton, 2014; Wang, Newport, & Hamilton, 2011) or emotion (Butler et al., 2016; Rauchbauer et al., 2015, Rauchbauer et al., 2016) on automatic imitation have used similarly abstracted associations of faces and hands to show significant effects, suggesting that anatomical plausibility is not essential for the modulation of automatic imitation by facial features.

Another potential limitation is that, while all studies examined the effect of emotional expressions on AI they varied in a number of ways including: the exact timing of stimuli presentation; the form of response cue; the number of actors viewed; and the nature of the response action. While we view this diversity as a strength given the consistency of results across studies, it is also possible that had the same measures been employed for all studies they would have revealed an effect that we missed. Similarly, it is possible that had we used a wider range of actors and expressions in experiments one, two and three we may have found a stronger effect as the results could not be modulated by idiosyncrasies in emotional expression. However, we would suggest that any effect that is so dependent on a particular time period or actor is unlikely to be a robust or theoretically interesting one when determining the role of imitation as a form of social signal.

Another important limitation is that the studies reported here focused only on one form of imitative behaviour, automatic imitation. Thus it is unclear that our findings can be reliably generalised to other forms of imitation, such as the behavioural mimicry, observed in a more naturalistic context (see Farmer et al., 2016 for a similar argument). To our knowledge to date there have been no direct studies on the effect of emotional expression on behavioural mimicry, possibly due to the difficulty of presenting emotional expressions in a controlled manner during a naturalistic encounter. One recent development that might allow the investigation of this question is the use of realistic virtual avatars to create a controlled but naturalistic interaction (Pan & de C Hamilton, 2015). In addition our findings do not preclude the large body of evidence demonstrating a close link between facial mimicry and the processing of emotional stimuli (Stel & Van Knippenberg, 2014; Tramacere, Ferrari, Gentilucci, Giuffrida, & De Marco, 2018).

### 8.3 Conclusion

In conclusion the current study investigated whether emotional expressions modulated stimulus-response compatibility effects for a range of motor responses. Across five studies we found no significant effect of emotional expression on SRCs even when the gestures being observed and executed had a strongly valenced meaning or when the study design and stimuli precisely matched those used in previous studies that had found an effect of emotional expression. This evidence for a null result was also supported by both a random effects meta-analysis and Bayesian *t*-tests. We combine our results with previous findings from the literature which suggest that emotional expressions have only a limited, if any role, in modulating SRCs.

## CRediT authorship contribution statement

**Harry Farmer:** Conceptualization, Methodology, Software, Formal analysis, Investigation, Resources, Data curation, Writing - original draft, Visualization, Project administration. **Raqeeb Mahmood:** Formal analysis, Investigation, Resources, Writing - review & editing. **Samantha E.A. Gregory:** Formal analysis, Investigation, Writing - review & editing. **Polina Tishina:** Formal analysis, Investigation, Writing - review & editing. **Antonia F. de Hamilton:** Conceptualization, Methodology, Writing - review & editing, Supervision, Funding acquisition.

## Appendix A Supplementary data

Supplementary material Image 1
