# Supplementary material for: Dynamic emotional expressions do not modulate responses to gestures
Source: Acta Psychol (Amst). 2021 Jan;212:103226. doi: 10.1016/j.actpsy.2020.103226 (PMC7755647; doi:10.1016/j.actpsy.2020.103226)
Supplement: Supplementary file 1 — Supplementary material [file mmc1.docx]

Dynamic Emotional Expressions Do Not Modulate Responses to Gestures: Supplementary Materials

# S1. Experiment 1

## S1.1. G*Power Sensitivity Analysis Protocol

**F tests -** ANOVA: Repeated measures, within factors

**Analysis:** Sensitivity: Compute required effect size

**Input:** α err prob = 0.05

Power (1-β err prob) = 0.8

Total sample size = 31

Number of groups = 1

Number of measurements = 4

Corr among rep measures = 0.5

Nonsphericity correction ε = 1

**Output:** Noncentrality parameter λ = 11.3871976

Critical F = 2.7058381

Numerator df = 3.0000000

Denominator df = 90.0000000

Effect size f = 0.2142805

## S1.2. Validation Ratings of Stimuli

**Table S1:** Means and standard deviations for the validation ratings (n = 20) of genuineness intensity and valance for each of the emotional faces used as stimuli in experiments 1 and 3. SD = Standard Deviation, Sig = uncorrected results of paired sample t-tests (within actor only) with same letters indicating a significant difference between conditions, * = *p* < .05, † = *p* < .01, ‡ = *p* < .001.

| **Actor** | **Emotion** | **Genuineness** | | | **Positivity** | | | **Intensity** | | |
| --- | --- | --- | --- | --- | --- | --- | --- | --- | --- | --- |
|  |  | Mean | SD | Sig | Mean | SD | Sig | Mean | SD | Sig |
| Female | Genuine Smile | 7.80 | 0.70 | a‡, b‡, c‡ | 7.80 | 1.15 | a‡, b‡, c‡ | 7.00 | 1.52 | a‡, b‡ |
|  | Polite Smile | 3.05 | 1.70 | a‡, d*,e† | 4.40 | 1.85 | a‡, d†, e‡ | 4.05 | 1.76 | a‡, c*, d† |
|  | Neutral | 4.75 | 2.22 | b‡, d* | 2.40 | 1.23 | b‡, d†, f† | 2.95 | 1.47 | b‡, c*, e‡ |
|  | Frown | 5.50 | 1.82 | c‡, e† | 1.55 | 0.83 | c‡, e‡, f† | 6.30 | 1.78 | d†, e‡ |
| Male | Genuine Smile | 6.60 | 2.19 | z‡, y* | 8.05 | 0.76 | z‡, y‡, x‡ | 6.45 | 2.04 | z†, y‡ |
|  | Polite Smile | 4.60 | 1.93 | z‡, x* | 5.55 | 1.00 | z‡, w‡, v‡ | 5.00 | 1.21 | z†, x†, w† |
|  | Neutral | 5.70 | 1.84 | y*, x* | 2.80 | 1.32 | y‡, w‡, u† | 3.35 | 2.06 | y‡, x†, v‡ |
|  | Frown | 5.00 | 2.34 |  | 1.75 | 1.16 | x‡, v‡, u† | 6.25 | 1.77 | w†, v‡ |

## S1.3. Analysis of Accuracy Results

To analyse accuracy, we calculated the percentages of trials which the participant responded correctly compared to those in which they either responded with an incorrect gesture or responded before 50ms or after 1000ms. These accuracy scores were then entered into a repeated measures ANOVA with actor, emotion and congruency as the IVs. There was no significant effect of actor, *F*(1, 30) = 0.29, *p =* .596, pƞ^2^ = .009. There was no significant effect of emotion, *F*(3, 90) = 1.37, *p* = .257, pƞ^2^ = .044. A significant effect of congruency was found, *F*(1, 30) = 8.39, *p* = .007, pƞ^2^ = .218, due to participants being more accurate in the congruent condition (EMM = 98.72%, SE = 0.42) compared to the incongruent condition (EEM = 97.27%, SE = 0.49). There was no significant interaction between actor and emotion *F*(3, 90) = 1.44, *p* = .236, pƞ^2^ = .046 or between emotion and congruency, *F*(3, 90) = 2.06, *p* = .112, pƞ^2^ = .064. However, there was a significant interaction between actor and congruency, *F*(1, 30) = 6.52, *p* = .016, pƞ^2^ = .178. Bonferroni corrected pairwise comparisons of the estimate marginal means (EMMs) indicated that this interaction was driven by the factor that for the male actor there was significantly greater accuracy in the congruent trials (EMM = 99.06%, SE = 0.33) than in the incongruent trials (EMM = 96.77%, SE = 0.56, *p* < .001, *d* = -.889) but for the female actor there was no significant difference between congruent (EMM = 98.39%, SE = 0.57) and incongruent (EMM = 97.78%, SE = 0.55, *p* = .337, *d* = -.195) trials. The three-way interaction was also non-significant, *F*(3, 90) = 2.17, *p* = .098, pƞ^2^ = .044.

# S2. Experiment 2

## S2.1. G*Power Sensitivity Analysis Protocol

**F tests -** ANOVA: Repeated measures, within factors

**Analysis:** Sensitivity: Compute required effect size

**Input:** α err prob = 0.05

Power (1-β err prob) = 0.8

Total sample size = 25

Number of groups = 1

Number of measurements = 4

Corr among rep measures = 0.5

Nonsphericity correction ε = 1

**Output:** Noncentrality parameter λ = 11.5119642

Critical F = 2.7318070

Numerator df = 3.0000000

Denominator df = 72.0000000

Effect size f = 0.2399163

## S2.2. Validation Ratings of Stimuli

**Table S2:** Means and standard deviations for the validation ratings (n = 20) of genuineness intensity and valance for each of the emotional faces used as stimuli in experiment 2. SD = Standard Deviation, Sig = uncorrected results of paired sample t-tests (between emotions within gaze direction and between each emotion and gaze direction) with same letters indicating a significant difference between conditions, * = *p* < .05, † = *p* < .01, ‡ = *p* < .001.

| **Gaze** | **Emotion** | **Genuineness** | | | **Positivity** | | | **Intensity** | | |
| --- | --- | --- | --- | --- | --- | --- | --- | --- | --- | --- |
|  |  | Mean | SD | Sig | Mean | SD | Sig | Mean | SD | Sig |
| Direct | Genuine Smile | 7.80 | 0.70 | a‡, b‡, c‡ | 7.80 | 1.15 | a‡, b‡, c‡ | 7.00 | 1.52 | a‡, b‡ |
|  | Polite Smile | 3.05 | 1.70 | a‡, d*,e† | 4.40 | 1.85 | a‡, d†, e‡, m* | 4.05 | 1.76 | a‡, c*, d† |
|  | Neutral | 4.75 | 2.22 | b‡, d* | 2.40 | 1.23 | b‡, d†, f† | 2.95 | 1.47 | b‡, c*, e‡ |
|  | Frown | 5.50 | 1.82 | c‡, e† | 1.55 | 0.83 | c‡, e‡, f† | 6.30 | 1.78 | d†, e‡ |
| Averted | Genuine Smile | 7.60 | 0.88 | z‡, y‡, x† | 8.15 | 0.81 | z‡, y‡, x‡ | 7.10 | 1.07 | z‡, y‡ |
|  | Polite Smile | 3.20 | 1.80 | z‡, w*, v* | 5.35 | 1.42 | z‡, w‡, v‡, m* | 4.50 | 1.47 | z‡, x†, w† |
|  | Neutral | 4.85 | 2.52 | y‡, w*, m‡ | 2.55 | 1.19 | y‡, w‡, u† | 3.10 | 2.08 | y‡, x†, v‡ |
|  | Frown | 5.45 | 2.16 | x†, v* | 1.50 | 1.05 | x‡, v‡, u† | 6.25 | 2.07 | w†, v‡ |

## S2.3. Analysis of Accuracy Results

To analyse accuracy, we calculated the percentages of trials which the participant responded correctly compared to those in which they either responded with an incorrect gesture or responded before 50ms or after 1000ms. These accuracy scores were then entered into a repeated measures ANOVA with gaze, emotion, and congruency as the IVs. There was a significant effect of gaze, *F*(1, 23) = 4.56, *p =* .044, pƞ^2^ = .165 because there was significantly higher accuracy in the direct gaze condition (EMM = 96.83%, SE = 0.56) compared to the averted gaze condition (EMM = 96.01%, SE = 0.66). There was also significant effect of emotion, *F*(3, 69) = 3.71, *p* = .016, pƞ^2^ = .139. Bonferroni corrected pairwise comparisons of the estimate marginal means did not reveal any significant differences between the different emotion conditions. However, there were two differences that approached significance (p < .1). First, there was greater accuracy for genuine smiles (EMM = 96.62%, SE = 0.67) compared to polite smiles (EMM = 95.23, SE = 0.78, *p* = .074, *d* = .390). Second there was greater accuracy for neutral expressions (EMM = 97.40, SE = 0.69) compared to polite smiles (*p* = .057, *d* = -.603). No significant effect of congruency was found, *F*(1, 23) = 1.6, *p* = .293, pƞ^2^ = .048. There was no significant interaction between gaze and emotion *F*(3, 69) = 1.28, *p* = .288, pƞ^2^ = .053, gaze and congruency, *F*(1, 23) = 0.62, *p* = .805, pƞ^2^ = .003 or emotion and congruency, *F*(3, 69) = 1.86, *p* = .144, pƞ^2^ = .075. The three-way interaction was also non-significant, *F*(3, 69) = 1.23, *p* = .306, pƞ^2^ = .051.

# S3. Experiment 3

## S3.1. G*Power Sensitivity Analysis Protocol

**F tests -** ANOVA: Repeated measures, within factors

**Analysis:** Sensitivity: Compute required effect size

**Input:** α err prob = 0.05

Power (1-β err prob) = 0.8

Total sample size = 30

Number of groups = 1

Number of measurements = 4

Corr among rep measures = 0.5

Nonsphericity correction ε = 1

**Output:** Noncentrality parameter λ = 11.4043194

Critical F = 2.7094021

Numerator df = 3.0000000

Denominator df = 87.0000000

Effect size f = 0.2179862

## S3.2. Analysis of Accuracy Results

To analyse accuracy, we calculated the percentages of trials which the participant responded correctly compared to those in which they either responded with an incorrect gesture or responded before 50ms or after 1000ms. These accuracy scores were then entered into a repeated measures ANOVA with actor, emotion and complementarity as the IVs. There was no significant effect of actor, *F*(1, 29) = 1.71, *p =* .202, pƞ^2^ = .056, emotion, *F*(3, 87) = 0.64, *p* = .594, pƞ^2^ = .021. or complementarity, *F*(1, 29) = 1.87, *p* = .182, pƞ^2^ = .060. There was no significant interaction between actor and emotion *F*(3, 87) = 0.96, *p* = .415, pƞ^2^ = .032, between actor and complementarity, *F*(1, 27) = 0.80, *p* = .378, pƞ^2^ = .027, or between emotion and complementarity, *F*(3, 87) = 0.31, *p* = .817, pƞ^2^ = .011. The three-way interaction was also non-significant, *F*(3, 87) = 0.53, *p* = .662, pƞ^2^ = .018.

## S3.3. Analysis of Handshakes by Laterality

At the suggestion of one of our reviewers we also ran an analysis of our data from this experiment that distinguished between the laterality of the observed hands. The logic of this analysis was that, because only right-hand handshakes are commonly observed in real life, a social modulation of complementarity might be stronger for actions made towards a right hand stimuli compared to a left hand stimuli.

Two repeated measures ANOVAs were conducted on the handshake complementarity reaction time data. The first took raw RTs as the DV and actor, emotion, observed hand laterality and complementarity as the IVs. There was a significant effect of actor, *F*(1, 29) = 10.40, *p* = .003, pƞ^2^ = .273, due to faster responses for the female actor (EMM = 498.74, SE = 15.54) compared to male actor (EEM = 507.88, SE = 14.20). A significant effect of emotion was found, *F*(3, 87) = 13.87, *p* < .001, pƞ^2^ = .324. Bonferroni corrected pairwise comparisons of the estimate marginal means indicated that this effect was due to significantly slower RTs in the neutral condition (EMM = 510.46, SE = 15.58) than in the genuine smile (EMM = 497.50, SE = 14.52, *p* < .001, *d* = -.157), polite smile (EMM = 503.74, SE = 14.55, *p* = .018, *d* = -.081) and frown (EMM = 501.52, SE = 14.80, *p* = .004, *d* = -.107) conditions. RTs for the polite smile were also significantly slower than for the genuine smile (p = .007, *d* = -.078). No other significant differences between emotional expressions were found. There was no significant effect of laterality, *F*(1, 29) = 0.31, *p* = .582, pƞ^2^ = .011. A significant effect of complementarity was also found, *F*(1, 29) = 10.19, *p* = .003, pƞ^2^ = .26, due to participants being faster to respond in the complementary condition (EMM = 497.5, SE = 14.23) compared to the uncomplementary condition (EEM = 509.14, SE = 15.60). There was no significant interaction between actor and emotion, *F*(3, 87) = 0.19, *p* = .902, pƞ^2^ = .007; between actor and laterality, *F*(1, 29) = 0.90, *p* = .766, pƞ^2^ = .003; between emotion and laterality, *F*(3, 87) = 0.61, *p* = .609, pƞ^2^ = .021; between actor and complementarity, *F*(1, 29) = 0.06, *p* = .810, pƞ^2^ = .002; between emotion and complementarity, *F*(3, 87) = 0.56, *p* = .642, pƞ^2^ = .019; or between laterality and complementarity, *F*(1, 29) = 1.96, *p* = .172, pƞ^2^ = .063. There was also no significant three-way interactions between actor, emotion and laterality, *F*(3, 87) = 0.84, *p* = .474, pƞ^2^ = .028; between actor, emotion and complementarity, *F*(3, 87) = 0.95, *p* = .421, pƞ^2^ = .032; between actor, laterality and complementarity, *F*(1, 29) = 1.57, *p* = .220, pƞ^2^ = .051; or between actor, laterality and complementarity, *F*(3, 87) = 0.349, *p* = .790, pƞ^2^ = .012. Finally, there was no significant four-way interaction between actor, emotion, laterality and complementarity, *F*(3, 87) = 0.89, *p* = .449, pƞ^2^ = .030.

The second ANOVA took PCompEs as the DV and actor, emotion, and laterality as the IVs. This analysis found no significant effect of actor, *F*(1, 29) = 0.08, *p =* .779, pƞ^2^ = .003; emotion, *F*(3, 87) = 0.50, *p =* .684, pƞ^2^ = .017; or laterality, *F*(1, 29) = 2.06, *p =* .162, pƞ^2^ = .066. There was no significant interaction between actor and emotion, *F*(3, 87) = 1.04, *p =* .381, pƞ^2^ = .034; between actor and laterality, *F*(1, 29) = 1.77, *p =* .194, pƞ^2^ = .058; or between emotion and laterality, *F*(3, 87) = 0.11, *p =* .956, pƞ^2^ = .004. Nor was there a significant three-way interaction between the IVs, *F*(3, 87) = 1.07, *p* = .365, pƞ^2^ = .036.

# S4. Experiment 4

## S4.1. G*Power Sensitivity Analysis Protocol

**F tests -** ANOVA: Repeated measures, within factors

**Analysis:** Sensitivity: Compute required effect size

**Input:** α err prob = 0.05

Power (1-β err prob) = 0.8

Total sample size = 43

Number of groups = 1

Number of measurements = 2

Corr among rep measures = 0.5

Nonsphericity correction ε = 1

**Output:** Noncentrality parameter λ = 8.2237646

Critical F = 4.0726538

Numerator df = 1.0000000

Denominator df = 42.0000000

Effect size f = 0.2186609

## S4.2. Validation Ratings of Stimuli

**Table S3:** Means and standard deviations for the averaged validation ratings (n = 20) of genuineness intensity and valance for each of the emotional expressions used as stimuli in experiment 4. SD = Standard Deviation, Sig = uncorrected results of paired sample t-tests (between emotions), * = *p* < .05, † = *p* < .01, ‡ = *p* < .001.

| **Emotion** | **Genuineness** | | | **Positivity** | | | **Intensity** | | |
| --- | --- | --- | --- | --- | --- | --- | --- | --- | --- |
|  | Mean | SD | Sig | Mean | SD | Sig | Mean | SD | Sig |
| Genuine Smile | 7.65 | 1.15 | ‡ | 8.15 | 0.95 | ‡ | 6.53 | 1.44 | ‡ |
| Frown | 5.35 | 1.68 |  | 1.40 | 0.55 |  | 7.43 | 1.13 |  |

# S5. Experiment 5

## S5.1. G*Power Sensitivity Analysis Protocol

**F tests -** ANOVA: Repeated measures, within factors

**Analysis:** Sensitivity: Compute required effect size

**Input:** α err prob = 0.05

Power (1-β err prob) = 0.8

Total sample size = 35

Number of groups = 1

Number of measurements = 3

Corr among rep measures = 0.5

Nonsphericity correction ε = 1

**Output:** Noncentrality parameter λ = 10.0720678

Critical F = 3.1316720

Numerator df = 2.0000000

Denominator df = 68.0000000

Effect size f = 0.2190028

## S5.1. Participants Ratings of Emotional Stimuli

### S5.1.1. Ratings Task Method

In order to assess and difference between participants perceptions of the still and dynamic stimuli participants viewed the genuine smile, neutral and frown expression videos/still images from each of the 16 actors and were asked to rate them for their genuineness, intensity and positivity on a scale from 1 to 9. Each rating was done in a separate presentation and a break was given midway through the task to allow participants to rest. Following this, still images of all the actors’ neutral expressions were displayed, and participants were asked to rate the actors faces on the characteristics of attractiveness, likability and trustworthiness again on a scale from 1 to 9. Data from the ratings task was average across the models in each stimuli type to provide a mean response for each question in each stimuli type (see Table S4 for means and SDs).

### S5.1.2. Ratings Task Results

To examine differences between evaluations of the dynamic and static emotional expressions three repeated measures ANOVAs were conducted taking stimuli type and emotion as the IVs and ratings of genuineness, intensity and positivity respectively as the DVs. For genuineness ratings there was no significant effect of stimuli type, *F*(1, 34) = 2.01, *p* = .167, pƞ^2^ = .059. There was a significant effect of emotion, *F*(2, 68) = 35.08, *p* < .001, pƞ^2^ = .508. Bonferroni corrected pairwise comparisons of the estimate marginal means indicated that this effect was due to a significantly higher ratings for the genuine smile (EMM = 6.69, SE = 0.17) compared to the frown (EMM = 4.08, SE = 0.27, *p* < .001) and neutral expression (EMM = 5.45, SE = 0.24, *p* = .002). The neutral expression was also rated as more genuine than the frown (*p* < .001). There was also a significant gaze by emotion interaction, *F*(2, 68) = 2.77, *p* < .001, pƞ^2^ = .23 (see Table S4 for post hoc t-tests). Pairwise comparisons suggested that this effect was driven by significantly higher genuineness ratings for genuine smiles in the dynamic compared to static conditions and significantly higher genuineness ratings in the static compared to dynamic condition for frowns (see Table S4 for means and SDs).

**Table S4:** Means and standard deviations for participant ratings (n = 35) of genuineness intensity and valance for the emotional faces used as stimuli in experiment 5 averaged across stimuli type. SD = Standard Deviation, Sig = uncorrected results of paired sample t-tests (between emotions within stimuli type direction and between each emotion and stimuli type) with same letters indicating a significant difference between conditions, * = *p* < .05, † = *p* < .01, ‡ = *p* < .001.

| **Stimuli Type** | **Emotion** | **Genuineness** | | | **Positivity** | | | **Intensity** | | |
| --- | --- | --- | --- | --- | --- | --- | --- | --- | --- | --- |
|  |  | Mean | SD | Sig | Mean | SD | Sig | Mean | SD | Sig |
| Dynamic | Genuine Smile | 6.45 | 1.21 | a*, b‡, m† | 7.55 | 0.79 | a‡, b‡ | 4.15 | 2.21 | a* |
|  | Neutral | 5.59 | 1.30 | a*, c‡ | 3.95 | 0.92 | a‡, c‡, m‡ | 3.61 | 1.38 | b‡, m‡ |
|  | Frown | 4.44 | 1.64 | b‡, c‡, n† | 2.33 | 0.86 | b‡, c‡, n‡ | 5.28 | 1.28 | a*, b‡, n‡ |
| Static | Genuine Smile | 6.93 | 1.07 | z‡, y‡, m† | 7.76 | 0.82 | z‡, y‡ | 4.22 | 2.25 | z‡, |
|  | Neutral | 5.32 | 1.72 | z‡, x‡ | 3.04 | 1.19 | z‡, x‡, m‡ | 4.53 | 1.58 | y‡,, m‡ |
|  | Frown | 3.72 | 1.89 | y‡, x‡, n† | 1.48 | 0.77 | y‡, x‡, n‡ | 7.75 | 1.11 | z‡, y‡, n‡ |

For positivity ratings there was a significant effect of stimuli type, *F*(1, 34) = 44.08, *p* < .001, pƞ^2^ = .565, Bonferroni corrected pairwise comparisons of the estimate marginal means indicated that this effect was due to a significantly higher ratings for the dynamic (EMM = 4.61, SE = 0.11) compared to static (EMM = 4.09, SE = 0.1) stimuli. There was also a significant effect of emotion, *F*(2, 68) = 590.48, *p* < .001, pƞ^2^ = .946. Bonferroni corrected pairwise comparisons of the estimate marginal means indicated that this effect was due to a significantly higher ratings for the genuine smile (EMM = 7.66, SE = 0.13) compared to the frown (EMM = 1.9, SE = 0.12, *p* < .001) and the neutral expression (EMM = 3.5, SE = 0.16, *p* < .001) and significantly higher ratings for the neutral expression compared to the frown (*p* < .001). There was also a significant interaction between stimuli type and emotion, *F*(2, 68) = 25.42, *p* < .001, pƞ^2^ = .428. Pairwise comparisons suggested that this effect was driven by significantly higher positivity ratings in the dynamic compared to static conditions for frowns and neutral expressions but not for smiles (see Table S4 for means and SDs).

For intensity ratings there was a significant effect of stimuli type, *F*(1, 34) = 125.27, *p* < .001, pƞ^2^ = .787, Bonferroni corrected pairwise comparisons of the estimate marginal means indicated that this effect was due to a significantly higher ratings for the static (EMM = 5.5, SE = 0.19) compared to dynamic (EMM = 4.35, SE = 0.19) stimuli. There was also a significant effect of emotion, *F*(2, 68) = 30.41, *p* < .001, pƞ^2^ = .472. Bonferroni corrected pairwise comparisons of the estimate marginal means indicated that this effect was due to a significantly higher ratings for the frown (EMM = 6.51, SE = 0.17) compared to the genuine smile (EMM = 4.18, SE = 0.37, *p* < .001) and the neutral expression (EMM = 4.07, SE = 0.24, *p* < .001). There was also a significant interaction between stimuli type and emotion, *F*(2, 68) = 46.11, *p* < .001, pƞ^2^ = .576. Pairwise comparisons suggested that this effect was driven by significantly higher intensity ratings in the static compared to dynamic conditions for frowns and neutral expressions but not for smiles (see Table S4 for means and SDs).

Finally, the average ratings of actors’ attractiveness, likeability and trustworthiness in the dynamic and still conditions was compared using three paired sample t-tests. There was no significant difference in ratings for attractiveness, *t*(34) = 0.89, *p* = .379, likeability, *t*(34) = -0.82, *p* = .416 or trustworthiness, *t*(34) = -0.43, *p* = .67.

## S5.2. Analysis of Accuracy Results

To analyse accuracy, we calculated the percentages of trials which the participant responded correctly compared to those in which they either responded with an incorrect gesture or responded before 50ms or after 1000ms. These accuracy scores were then entered into a repeated measures ANOVA with stimuli, emotion and congruency as the IVs. There was a significant effect of stimuli type, *F*(1, 34) = 5.55, *p =* .024, pƞ^2^ = .140, due to participants being more accurate in the dynamic condition (EMM = 96.15%, SE = 0.47) compared to the static condition (EEM = 94.85%, SE = 0.63). There was no significant effect of emotion, *F*(2, 68) = 0.10, *p* = .904, pƞ^2^ = .003. A significant effect of congruency was found, *F*(1, 34) = 59.41, *p* < .001, pƞ^2^ = .636, due to participants being more accurate in the congruent condition (EMM = 98.05%, SE = 0.28) compared to the incongruent condition (EEM = 92.95%, SE = 0.78). There was no significant interaction between stimuli type and emotion *F*(2, 68) = 1.32, *p* = .275, pƞ^2^ = .037, between stimuli type and congruency, *F*(1, 34) = 3.53, *p* = .069, pƞ^2^ = .094, or between emotion and congruency, *F*(2, 68) = 0.69, *p* = .505, pƞ^2^ = .020. The three-way interaction was also non-significant, *F*(2, 68) = 0.37, *p* = .690, pƞ^2^ = .011.
